# Discovery of α-Amidobenzylboronates as Highly Potent Covalent Inhibitors of Plasma Kallikrein

**DOI:** 10.1021/acsmedchemlett.3c00572

**Published:** 2024-03-28

**Authors:** Matthew Allison, Rebecca L. Davie, Adrian J. Mogg, Sally L. Hampton, Jonas Emsley, Michael J. Stocks

**Affiliations:** † Biodiscovery Institute, School of Pharmacy, University of Nottingham, Nottingham, NG7 2RD, United Kingdom; ‡ KalVista Pharmaceuticals Limited, Salisbury, SP4 0BF, United Kingdom

**Keywords:** Plasma kallikrein, Serine protease, Covalent inhibitor, Boronates

## Abstract

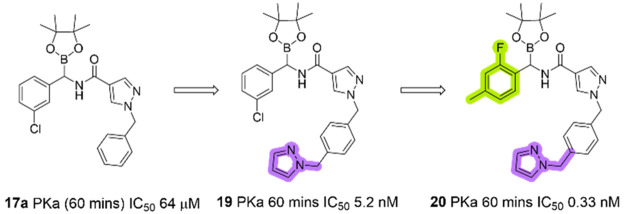

Hereditary angioedema (HAE), a rare genetic disorder, is associated with uncontrolled plasma kallikrein (PKa) enzyme activity leading to the generation of bradykinin swelling in subcutaneous and submucosal membranes in various locations of the body. Herein, we describe a series of potent α-amidobenzylboronates as potential covalent inhibitors of PKa. These compounds exhibited time-dependent inhibition of PKa (compound **20** IC_50_ 66 nM at 1 min, 70 pM at 24 h). Further compound dissociation studies demonstrated that **20** showed no apparent reversibility comparable to d-Phe-Pro-Arg-chloromethylketone (PPACK) (**23**), a known nonselective covalent PKa inhibitor.

Plasma prekallikrein (PK) is a serine protease which circulates as a bound complex with its substrate, high-molecular-weight kininogen (HK).^1^ PK has structural homology to factor XI (FXI), with both containing four apple domains at the *N*-terminus.^2^ The PK–HK complex in plasma^3^ can be activated by a single cleavage from coagulation factor XIIa (FXIIa) resulting in the activated form, plasma kallikrein (PKa). PKa performs a double cleavage of high-molecular-weight kininogen (HK) to liberate the vasoactive peptide bradykinin, which binds the B2 bradykinin receptors (B2R) on endothelial cells, leading to activation of signaling events causing vascular permeability and an inflammatory response (Figure 1).

**Figure 1:**
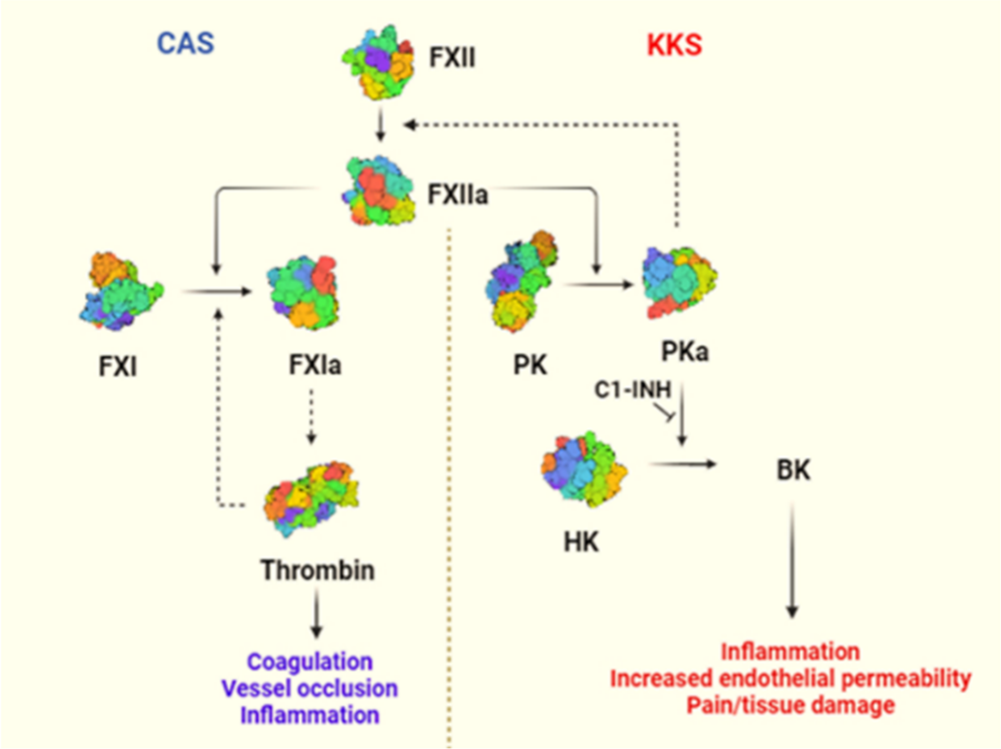
Schematic of (left) contact activation system (CAS) and (right) kallikrein–kinin system (KKS). Both initiated by factor XIIa, the cascade leading to bradykinin production and the positive feedback mechanism within the KKS are shown.

B2R is an essential G protein-coupled receptor (GPCR) involved in regulating homeostasis of the cardiovascular system as a vasodepressor.^4^ Binding of bradykinin to B2R promotes vasodilation, increased endothelial permeability, and capillary bed leakage, which in severe cases of bradykinin overproduction displays the symptoms of an acute angioedema attack.^5^ Accordingly, significant interest has developed around PKa as a drug target for the treatment of bradykinin-mediated angioedema such as hereditary angioedema (HAE). HAE is a rare disorder, occurring in three main subtypes; type I (quantitative) and type II (qualitative) originate from a deficiency and dysfunction in the endogenous C1 inhibitor protein (C1-INH), respectively, due to mutations of the C1-INH *SERPING*1 gene. Type III occurs as a result of mutation on the factor XII gene, which affects the PKa-mediated BK formation.^6^

C1-INH is the endogenous inhibitor of PKa, FXIIa and other proteases in the plasma, and HAE patients with reduced C1-INH functional levels are unable to effectively block PKa enzyme activity. Intravenously administered recombinant human C1-INH as well as plasma-derived C1-INH (pdC1-INH) have delivered safe and efficacious treatments of HAE attacks. HAE facilitates spontaneous and uncontrolled activation of the KKS.^7^ Patients have the potential to exhibit symptoms, such as subcutaneous and laryngeal swelling, and can also be accompanied by severe abdominal pain and obstruction of major organs.^8^

The PKa inhibitors berotralstat **1** is approved for prophylactic use and sebetralstat **2**(9) is in late-stage clinical studies for the acute on-demand treatment of HAE attacks (Figure 2). Studies with sebetralstat have demonstrated that pharmacological inhibition of PKa suppresses the KKS, resulting in suppression of both the cleavage of the PKa substrate HK and the feedback activation of the KKS in plasma, thereby inhibiting the generation of bradykinin.^10,11^ Approved acute treatments of HAE attacks involve injected or infused therapies and include icatibant (Firazyr) and C1-INH (Ruconest, Berinert). Berotralstat (Orladeyo) is the only approved oral therapy acting as an inhibitor of PKa for the prophylactic treatment of HAE.^12^ Clinical studies showed a 150 mg daily dose of berotralstat is efficacious as an oral prophylactic for HAE.^12^ Other HAE therapies such as icatibant (Firazyr), a peptidomimetic injectable drug, is indicated in the EU for the symptomatic treatment of acute attacks in patients with HAE.^13^ While other therapies treat the symptoms of acute attacks in patients with HAE through antagonistic suppression of the B2R, there is a need for further highly effective oral prophylactic treatments to address the outbreak of angioedema events.^14^

**Figure 2:**
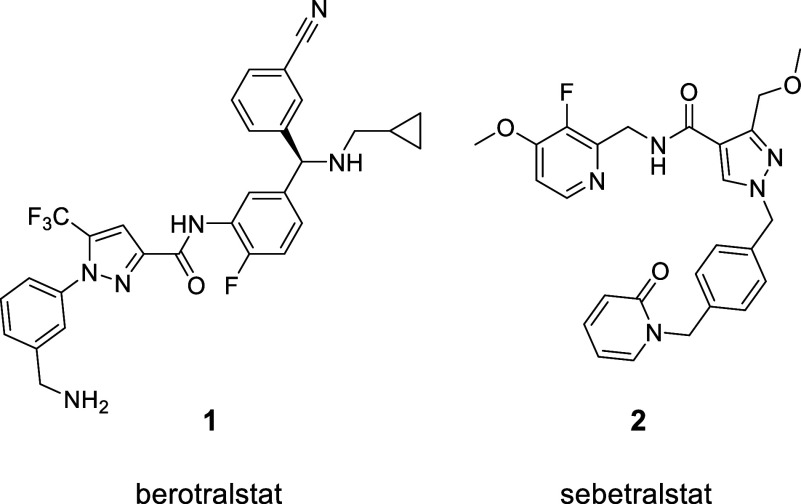
Chemical structures of berotralstat and sebetralstat.

Increasingly, boron-containing molecules^15,16^ are emerging as structures that target key nucleophilic residues within biological targets, promoting the formation of a covalent bond to maximize potency and elicit prolonged physiological responses, compared with conventional reversible inhibitors (Figure 3).^17−19^

**Figure 3:**
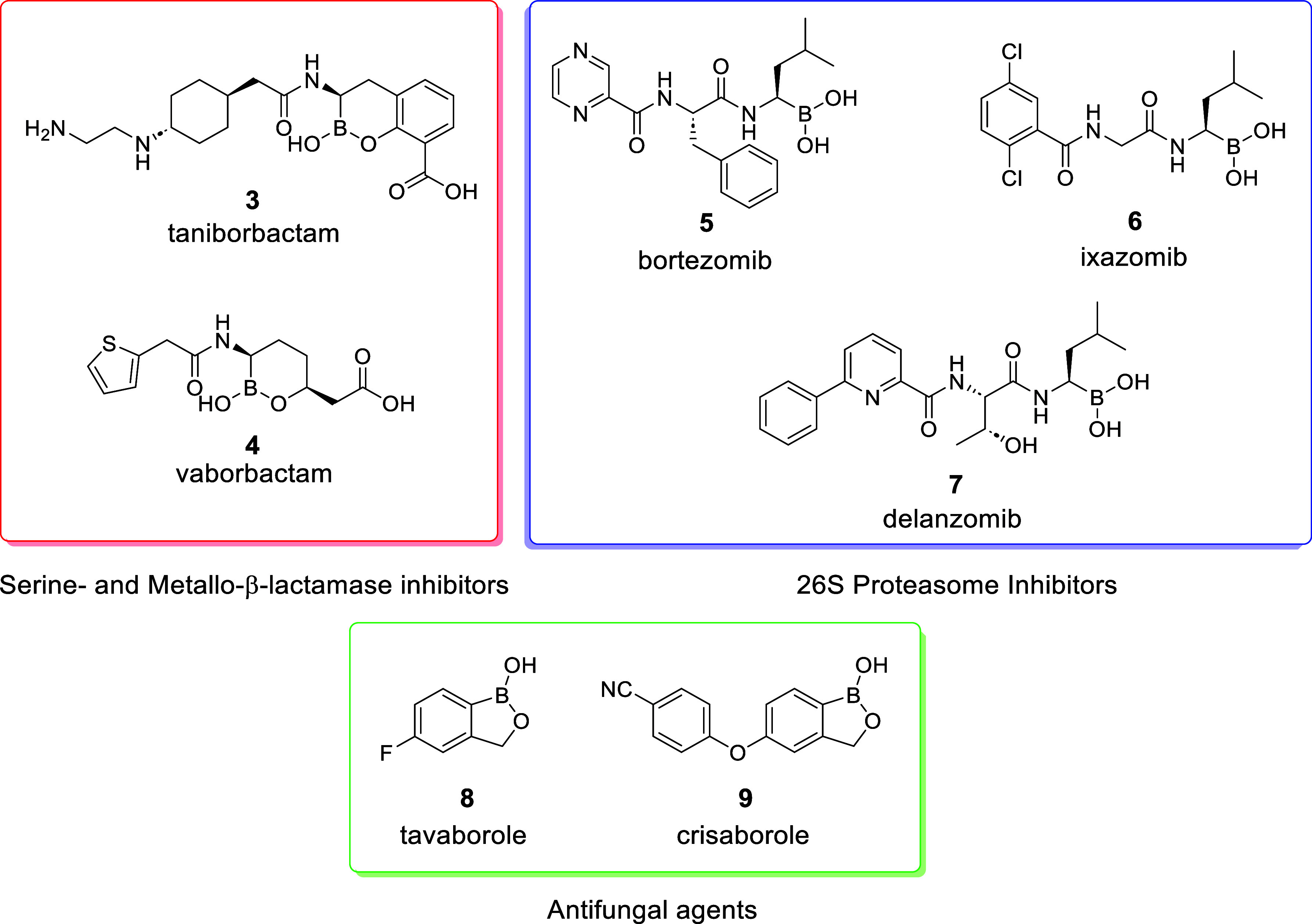
Examples of literature boron-containing compounds under FDA approval or in clinical development.

Taniborbactam **3** and vaborbactam **4** possess boronic ester motifs designed to covalently inhibit serine β-lactamases as a combination therapy alongside the administration of antibiotics. This is a strategy not yet seen in the design of clinical candidates targeting PKa. Lewis acids, such as boron, possess a vacant p-orbital, capable of accepting a lone pair of electrons to generate a stable borate anion. Interestingly, this signifies that, while capable of undergoing covalent interactions with nucleophilic residues, the nature of this interaction is reversible, which may be beneficial to the toxicology profile of such inhibitors, with off-target toxicity being a frequent concern for covalent inhibitors.^19^

Bortezomib **5** is an FDA-approved inhibitor of the 26S proteasome used for the treatment of various cancers, operating via the covalent interaction of the boronic acid moiety with the active site of the proteosome and showing high potency (IC_50_ = 2.4 nM) in in vitro cellular assays.^20,21^ Taniborbactam **3** is under clinical development as a combination therapy for the treatment of carbapenem-resistant bacterial infections via the inhibition of serine- and metallo-β-lactamases. Likewise, it functions via the covalent interaction of key enzyme residues with the boron center, with IC_50_ potency ranging from 5 to 490 nM over a range of bacterial species within a whole-cell assay.^22^ Crisaborole **9**, used for topical treatment of fungal infections, is an NSAID inhibitor of the fungal phosophodiesterase 4 (PDE4) enzyme, achieving 490 nM potency with similar inhibition against the release of cytokines TNF-α, IL-2, and IFN-γ with high selectivity.^23^ Demonstrably, there is significant value emerging from the incorporation of boron warheads into pharmaceutical agents, translating to potential for high efficacy.^24,25^

The discovery and development of PKa inhibitors has recently been extensively reviewed.^26^ However, to the best of our knowledge, the only boronic-acid-containing PKa inhibitor to date is compound **10** (PKa IC_50_ 45 nM), a nonselective compound that is also reported to be an inhibitor of FXIIa (IC_50_ 190 nM), trypsin (IC_50_ 52 nM), plasmin (IC_50_ 394 nM), and FXIa (IC_50_ 13 nM) (Figure 4).^27^

**Figure 4:**
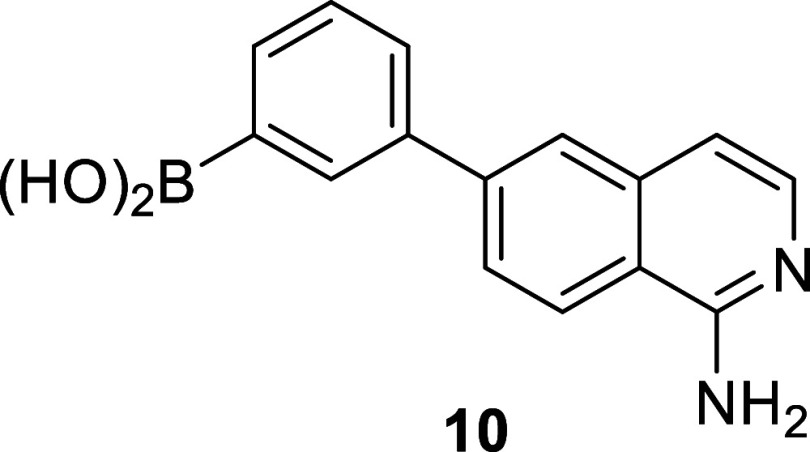
Chemical structure of the only known nonselective boronic acid containing PKa inhibitor compound **10**.

Applying this covalent inhibitor design approach to PKa presented the challenge of positioning the warhead appropriately to ensure productive interaction with the catalytic serine of the Ser195, His57, Asp102 catalytic triad.^28,29^ This was explored through a dual pocket binding approach, gaining inspiration from a literature PKa inhibitor **11** whose binding mode spans the S1 and S4 binding pockets within the PKa active site.^30^

Compound **11** (Figure 5),^31^ a competitive, reversible inhibitor of PKa (IC_50_ = 2 nM) was of particular interest due to the positioning of Ser195 relative to the main scaffold. This sparked interest in the installation of a boron warhead on the benzylic position of analogues of **11** to induce a potential covalent interaction with Ser195 within the binding site of PKa.

**Figure 5:**
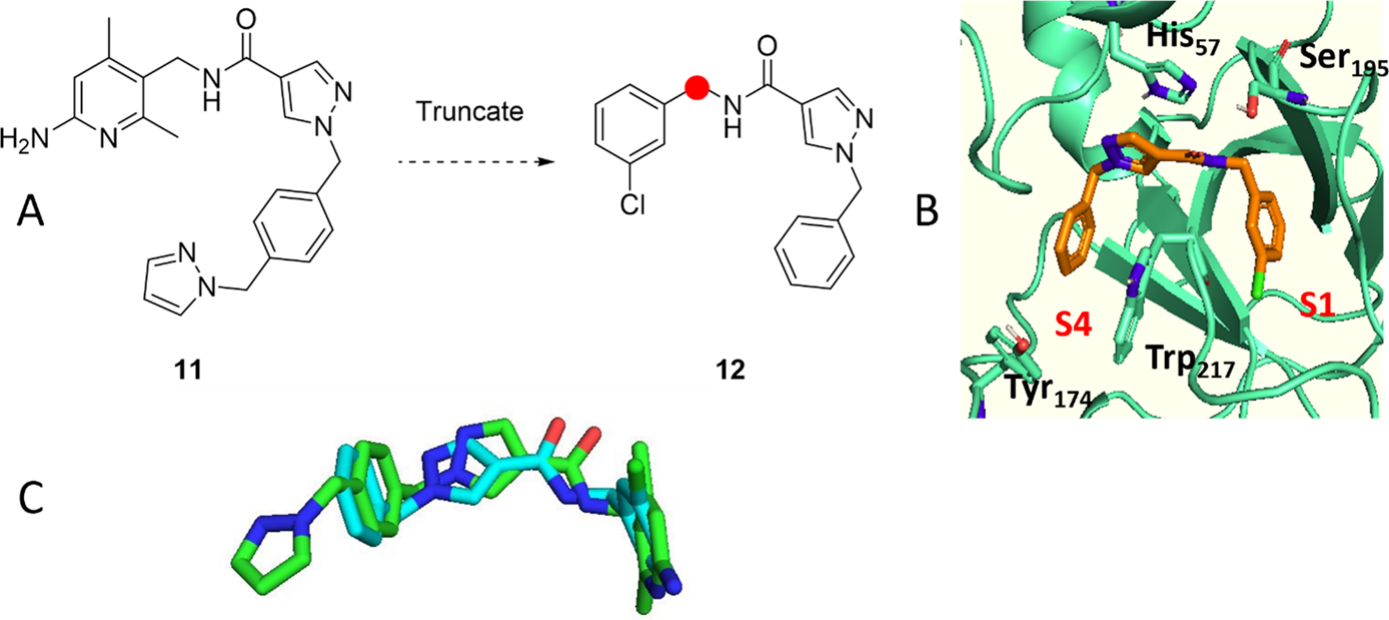
(A) Truncation of **11** to the structure of interest **12**. (B) Docking of **12** into PKa active site (PDB 6O1S) with key residues shown in bold.^30^ (C) Overlay of docked structure of **11** (teal) with **12** (green) in PKa. Docking experiments were performed using OEDOCKING Hybrid docking.^32^

In initial studies, we investigated a truncated fragment of **11**, omitting the terminal methylpyrazole unit and replacing the aminopyridine S1 group with the nonbasic 3-chlorophenyl group. These changes were carried out to enhance ease of synthesis and compound stability due to possible incompatibility between boronates and nucleophilic groups, such as the amine on the aminopyridine S1 group.

Molecular docking of truncated fragment **12** into the PKa active site showed comparable positioning of the benzylic carbon, extending from the S1 pocket, suggesting **12** to be a suitable candidate to bear a covalent warhead and for subsequent structure–activity relationship (SAR) investigations. Herein, the synthesis and isolated-enzyme potency data for a series of novel sub-nM PKa inhibitors are reported.

Initial synthetic explorations focused on the installation of a boron warhead at the highlighted benzylic position of **12**. This was achieved by performing a Matteson homologation sequence of arylboronic pinacol esters **13a**–**b** to yield the corresponding α-chloroboronate intermediates **14a**–**b**, followed by displacement with LiHMDS and acidic silyl deprotection to afford the corresponding α-aminoboronate hydrochloride salts **15a**–**b** (Scheme 1).^33,34^

**Scheme 1:**
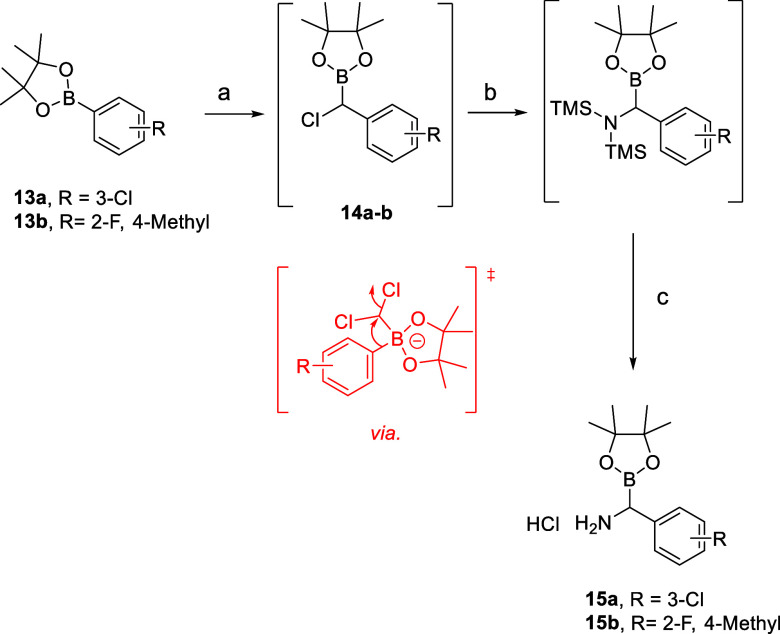
Synthesis of Compounds **15a**–**b** Reagents and conditions: a) *n*-BuLi, CH_2_Cl_2_, THF, −78 °C, 16 h; b) LiHMDS (1 M in THF) THF, −78 °C, 16 h; c) 4 N HCl in dioxane, Et_2_O, −60 °C, 3 h. Yield: 40–45% (3 steps).

Amidation of **15a** with **16a** gave access to α-amidoboronate **17**, and the corresponding boronic acid **18** was obtained under mild conditions via biphasic transesterification with pentylboronic acid (Scheme 2).^35^

**Scheme 2:**
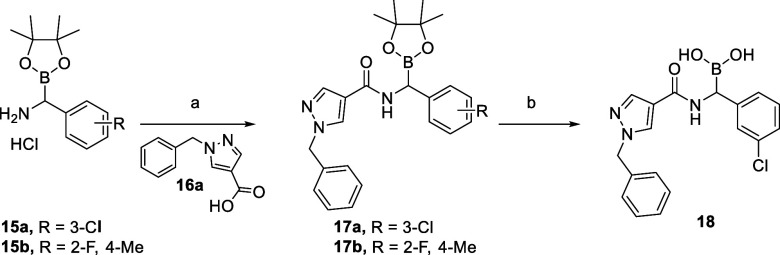
Synthesis of Compound **18** Reagents and conditions: a) **16a**, HATU, DIPEA, MeCN, 0 °C to rt, 3 h, 55–58%; b) 3 N HCl, C_5_H_11_B(OH)_2_, MeOH/cyclohexane (1:1), rt, 16 h, 23%.

Isolated enzyme potency was determined against PKa and FXIIa, using FXIIa as a test for the selective binding interaction with PKa. IC_50_ measurements were calculated following enzyme and inhibitor preincubation over a 60 min time-course to elucidate possible covalent binding character, under the premise that observed compound potency should increase over time in the case of a covalent inhibitor due to increasing active site occupancy (Table 1).

**Table 1:** Biological Activity of Compounds **15a**, **17a**, and **18** against Plasma Kallikrein and FXIIa

	IC_50_ (μM)^a^			
	PKa		FXIIa	
Example	5 min	60 min	5 min	60 min
**15a**	>400	>400	>400	>400
**17a**	>400	64	>400	>400
**17b**	2.3	0.033	>400	>400
**18**	>400	>400	>400	>400

a Data are expressed as mean of 2 experiments, where each experimental curve was performed in triplicate.

Surprisingly, **18** showed no activity against either enzyme; after consideration, this was thought to be likely due to its instability in DMSO and aqueous media, causing protodeborylation, a process seen during purification and observed by LCMS. Likewise, intermediate **15a**, also unstable over time in DMSO, showed no activity. Curiously, **17a** demonstrated activity (64 μM at 60 min) after no initial observation of enzyme activity at 5 min preincubation, whereas **17b** showed improved activity at both time points. These initial results provided promise for the exploration of further analogues.

Following these findings, corresponding analogues of **17a** and **17b** with the extended S4 pyrazole, bearing structural similarity to **10** were synthesized along with further S1 and S4 variants (Scheme 3). Amidation of the α-aminoboronate hydrochloride salts proceeded well to give the **19**–**22**. The low yields obtained generally reflect the difficulty of purification and tendency of these compounds to decompose in reverse-phase purification media via cleavage of the pinacol ester and subsequent protodeborylation of the boronic acid as observed by LCMS analysis of purified fractions. Along with the 3-chlorophenyl S1 substituent, the 2-fluoro-4-methylphenyl analogue was investigated, due to known high affinity for the PKa S1 binding site and their lack of nucleophilicity compared with more conventional S1 groups of this type, such as aminopyridine.^30^

**Scheme 3:**
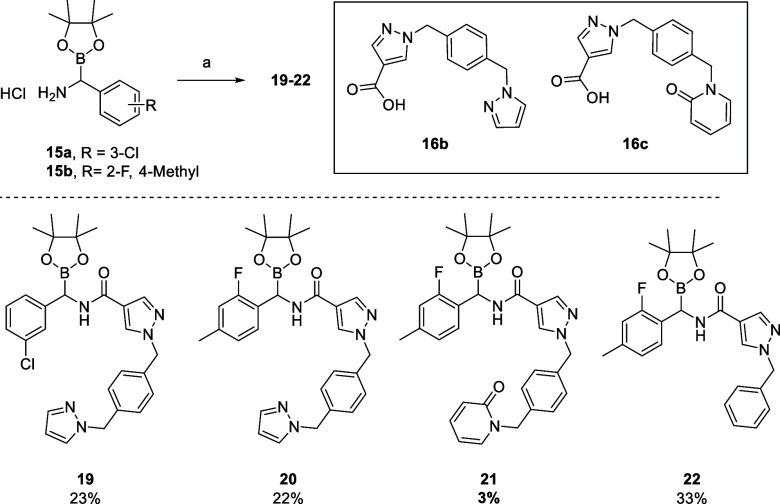
Synthesis of Compounds **19**–**22** Reagents and conditions: a) carboxylic acids **16a**–**c** HATU, DIPEA, MeCN, 0 °C to rt, 3 h; for **16b**, see ref (36); for **16c**, see ref (9).

Compounds **19**–**22** showed much improved biological activity through a combination of improved S1 binding (**17a** vs **22**) and elongation of the S4 fragment to include a methylpyrazole or methylpyridone unit (**19**–**21**), thought to undergo a key S4 π-stacking interaction with Tyr174 (Table 2).

**Table 2:** Biological Activity of Compounds **19**–**27** and **29**–**32** against Plasma Kallikrein

	PKa IC_50_ (nM)^a^		
Example	1 min	10 min	60 min
**19**	255	46	5.2
**20**	66	6.9	0.3
**21**	94	17	1.9
**22**	2,307	152	33
**23**	86	15	2.1
**24**	>40,000	>40,000	>40,000
**25**	270	284	278
**26**	199	203	196
**27**	37	37	35
**29**	>40,000	>40,000	>40,000
**30**	>40,000	7,263	5,975
**31**	1,534	2,173	2,637
**32**	41	41	34

a Data are expressed as mean of 2 experiments, where each experimental curve was performed in triplicate.

Occupation of the S4 and S1 pockets may have the effect of “anchoring” the scaffold into place, enabling productive interaction between the boron warhead and Ser195. In the case of each compound (**19**–**22**), a clear trend of increasing biological activity against time was visible, indicative of covalent binding, with the highest activity being observed for **20** (0.33 nM at 60 min preincubation time). Interestingly, **20** had no effect on the inhibition of FXIIa activity over similar time course experiments, showing a propensity for high PKa selectivity. Compound **23** is a known nonselective covalent inhibitor of PKa^37^ and was selected as a covalent control for method validation.
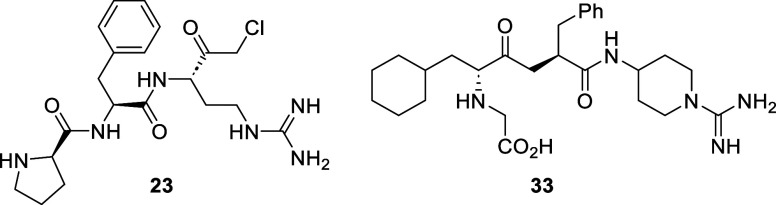


The isolated enzyme activity of **20** was seen to continually increase over 24 h, achieving an IC_50_ value of 0.29 nM at 2 h and 0.07 nM at 24 h (Figure 6). This suggests that once bound within PKa, **20** forms a stable complex with the protein without the propensity to protodeborylate or dissociate from PKa.

**Figure 6:**
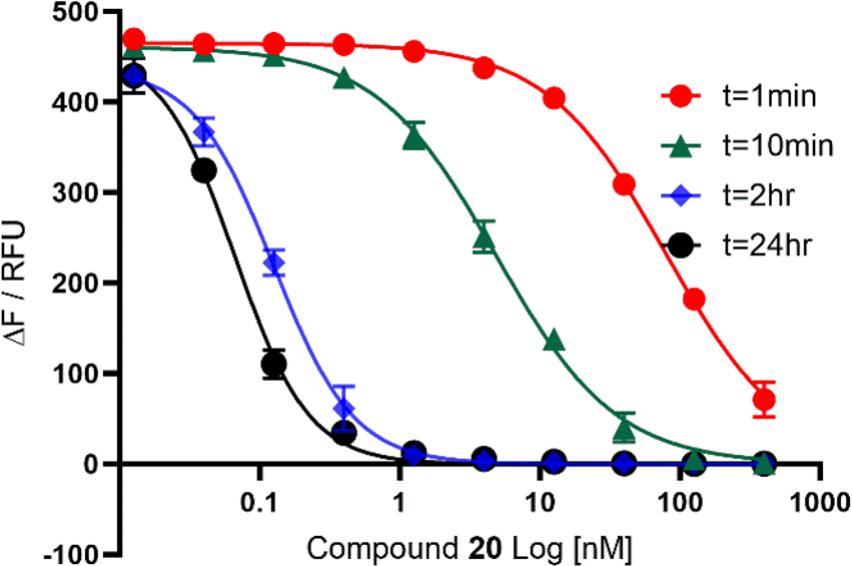
Extended time course for **20** against plasma kallikrein. Δ*F* = change in fluorescence, and RFU = relative fluorescence units.

To further assess the covalent binding character of **19**–**22**, a series of matched pairs **24**–**27**, omitting the boronate warheads, were synthesized (Scheme 4).

**Scheme 4:**
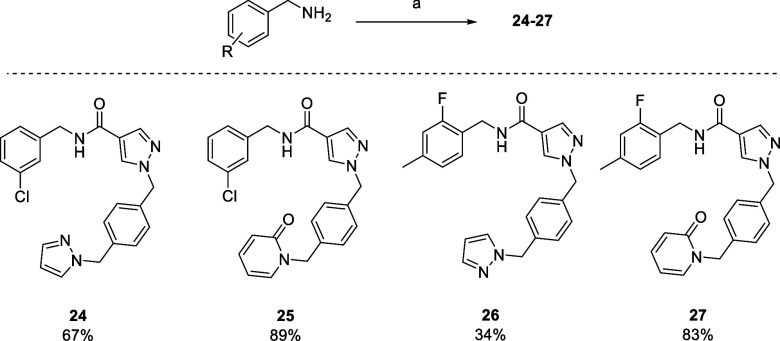
Synthesis of Compounds **24**–**27** Reaction conditions: a) carboxylic acids **16b**–**c**, HATU, DIPEA, MeCN, 0 °C to rt, 3 h.

No significant change in activity was observed over the 1-hour time course experiments for **24**–**27**, indicating reversible, noncovalent inhibitors of PKa. Compound **24** showed no PKa inhibition, whereas **25** and **26** showed comparable potency of 278 and 196 nM after 1 h preincubation time, respectively. Compound **27** showed the highest activity of this series, showing an IC_50_ of 35 nM after a 1-hour preincubation (Table 2).

Overall, this study showed that **24**–**27** exhibit significantly lower biological activity against PKa in comparison to **19**–**22**, as well as demonstrating noncovalent binding over the 1-hour time course experiments, providing further evidence that compounds **19**–**22** bind covalently to PKa.

Due to the limitations encountered in the synthesis and purification of the α-amidobenzylboronates (**19**–**22**), a second series of boronate-containing compounds was explored, where the amide group was replaced by a ketone to give compounds **31** and **32**. The compounds were prepared through the reaction of pinacol diboronate with intermediates **29** and **30**,^38^ synthesized from common intermediate **28** through an Aldol reaction with commercial benzaldehydes (Scheme 5).

**Scheme 5:**
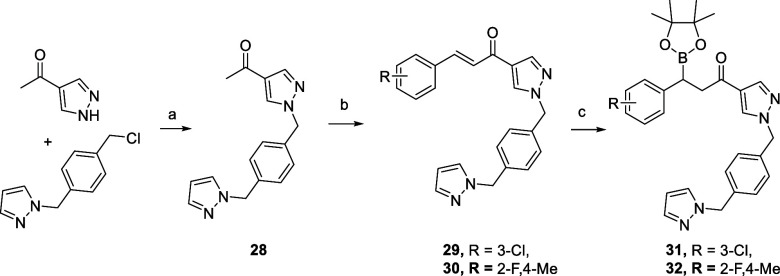
Synthesis of Compounds **28**–**32** Reagents and conditions: a) K_2_CO_3_, DMF, rt, 80%; b) 3-Cl benzaldehyde or 2-F, 4-Me benzaldehyde, NaOH, methanol, rt, 3 h, 70–81%; b) B_2_Pin_2_, DPEPhos (0.1 mol %), CuCl (0.03 mol %), KO^t^Bu, MeOH, THF, rt, 16 h, 34–56%.

Intermediates **29**–**30** showed very weak or no inhibition of PKa, and this may be a result of either the geometry of the exit vector from the S1 binding pocket imposed by the rigid alkene linking group or the removal of a key hydrogen bonding interaction observed between the amide N–H and the protein (Ser597). In contrast, compounds **31** and **32** demonstrated reasonable levels of PKa activity. However, they did not display the time course activity exhibited by **19**–**23**, suggesting noncovalent reversible inhibition, with **32** having an IC_50_ of ∼40 nM, throughout the time course experiment (Table 2).

Further covalent binding mechanistic evaluation was performed using compound dissociation assay methodology.^39−41^ Boronates **19**–**20**, covalent reference inhibitor **23**, and compounds **24** and **26** were subjected to a 62.5-fold dilution after 10 min preincubation, and the inhibitor dissociation was monitored using the fluorogenic substrate.^42^ It was shown that **23** and **20** showed no apparent reversibility over the 600 s time frame of the kinetic dissociation experiment, suggesting that they are covalent inhibitors of PKa. Interestingly, **19** showed slow dissociation over the time course, suggesting possible reversible covalent inhibition, whereas **24** and **26** showed rapid dissociation from PKa. In the same experiment, boronate **32** displayed slow dissociation over the experimental time course, whereas compound **31** demonstrated fast dissociation from PKa. The known highly potent reversible PKa inhibitor FE99026 **33** (IC_50_ ∼ 4–6 nM),^43^ demonstrated slow dissociation from PKa (Figure 7).

**Figure 7:**
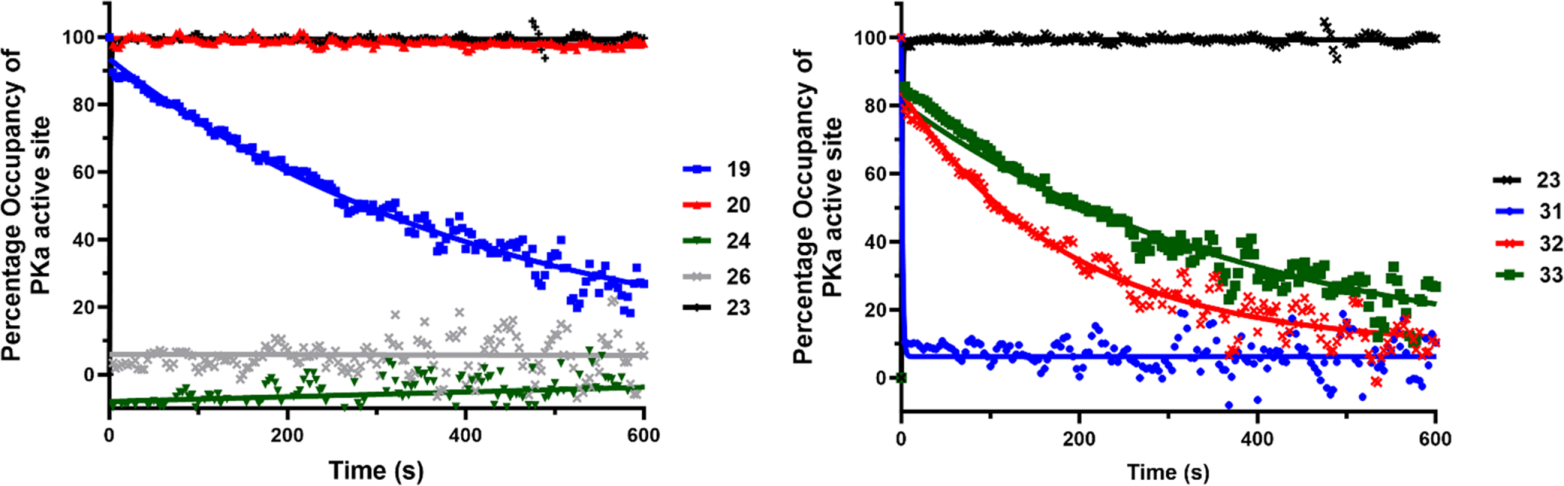
LHS Dissociation curves of **19**–**20**, covalent control **23**, and compounds **24** and **26**. RHS Dissociation curves of covalent control **23**, compounds **31**–**32**, and noncovalent control **33**.

Time-dependent potency determination was carried out against FXIa, thrombin, trypsin, and plasmin to further evaluate the selectivity of compounds (Table 3). FXIa shares the closest structural homology with PKa of all trypsin like proteases with 58% similarity in sequence identity.^44^ Selected compounds were assayed against FXIa alongside PKa, and all compounds showed a distinct preferential activity toward PKa over FXIa. Compound **20** showed modest inhibitory activity of FXIa, achieving an IC_50_ of 12,230 nM after 60 min preincubation time. In addition, inhibitory potency was seen to increase at each time point, suggesting a mild covalent interaction within the FXIa active site. Compound **20** also showed some time-dependent inhibition against plasmin, achieving an IC_50_ of 12,090 nM after 60 min preincubation time.

**Table 3:** Compound Selectivity against FXIa, Thrombin, Trypsin, and Plasmin

	FXIa^a^			Thrombin^a^			Trypsin^a^			Plasmin^a^		
Example	1 min	10 min	60 min	1 min	10 min	60 min	1 min	10 min	60 min	1 min	10 min	60 min
**19**	IA	IA	IA	IA	IA	IA	IA	IA	IA	IA	IA	IA
**20**	18,770	16,470	12,230	IA	IA	IA	IA	IA	IA	22,010	19,810	12,090
**24**	IA	IA	IA	IA	IA	IA	IA	IA	IA	IA	IA	IA
**26**	IA	IA	IA	IA	IA	IA	IA	IA	IA	IA	IA	IA
**31**	IA	IA	IA	IA	IA	IA	IA	IA	IA	IA	IA	IA
**32**	IA	IA	IA	IA	IA	IA	IA	IA	IA	IA	IA	IA
**23**	IA	IA	IA	3,550	981	187	484	165	28	3,639	1,222	248

a Data are expressed as mean of 2 replicates from *n* = 1 experiments. IA IC_50_ > 40,000 nM

In all cases, enzyme potency was seen to have a >1000-fold preference toward PKa over FXIa, thrombin, trypsin, and plasmin. For FXIa, this selective behavior could reflect the difference in structure of the P4 sites between PKa and FXIa with Tyr174 in PKa being substituted for a Glu residue in the case of FXIa,^45^ thus disenabling a key π-stacking interaction in the P4 binding site. PPACK **23**, was shown to have time-dependent inhibition against thrombin, trypsin, and plasmin.

It was demonstrated that compounds **19**–**22** covalently inhibit PKa through analysis of time-dependent binding kinetics and through comparison of activity with homologues **24**–**27**. Compound **20** displayed the most potent binding activity against PKa, showing picomolar activity at 24 h. In the compound dissociation assay, **20** demonstrated no apparent reversibility, suggesting that **20** may be a covalent inhibitor. Additionally, high PKa selectivity for **20** was observed against FXIa, thrombin, trypsin, and plasmin.

Molecular docking experiments were conducted to visualize the potential binding mode of **20**. However, it soon became apparent that the pinacol boronate group was too large to fit into the Ser195 subpocket. Having previously experienced hydrolysis of the pinacol boronate during purification, model substrate **17b** was used to study the stability of the pinacol boronate in PBS buffer by NMR (see Supporting Information Figure S1). **17b** was rapidly hydrolyzed to pinacol plus the boronic acid **35**, in which **35** existed in equilibrium with the oxaborolane **34** (Scheme 6), an observation recently reported for the synthesis of aminoboronic acids as LONP1 inhibitor compounds.^46^

**Scheme 6:**
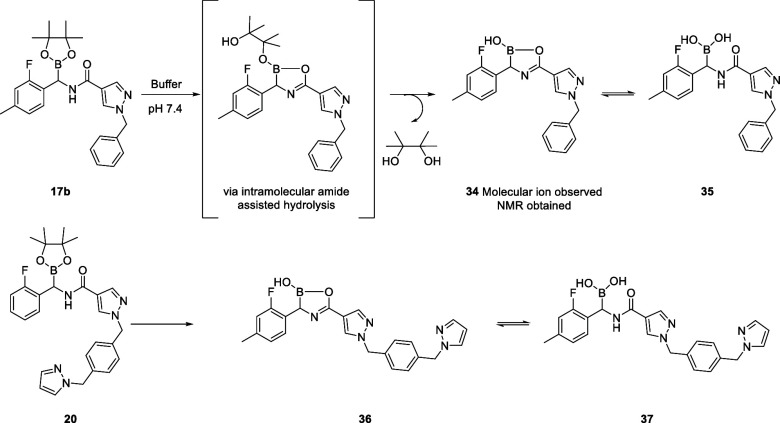
Hydrolysis of **17b** to **34** and **35** in pH7.4 Phosphate Buffer and Proposed Hydrolysis of **20** to **36** and **37**

A docking study was performed on oxaborolane **36** and boronic acid **37**, through docking into the Ala-195 mutant, followed by visualization of the docking results in PKa. Compound **37** showed interaction with Ser195, whereas **36** did not bind (Figure 8).

**Figure 8:**
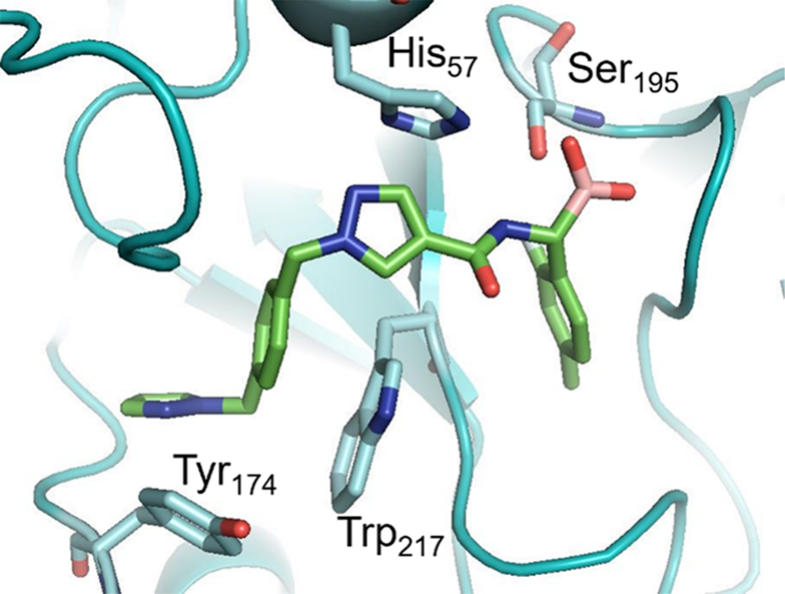
Docking of boronic acid **37** into the PKa active site (Ala-195 mutant). Docking results were subsequently visualized in PKa. Docking experiments were performed using OEDOCKING Hybrid with results visualized in PKa active site (PDB 6O1S) using the PyMOL Molecular Graphics System, Version 2.0 Schrödinger, LLC.

A docking study was performed with ketone **32** as assisted hydrolysis via ketone enolization, which would be disfavored due to the presence of the electropositive β-boronate. In this case, docking results showed **32** bound to the PKa active site with the pinacol boronate exposed to solvent, giving further rationalization for the noncovalent interaction of **32** (see Supporting Information Figure S2).

The novel class of α-amidobenzylboronates is the first reported class of covalent inhibitors targeting plasma kallikrein. Their discovery adds to the available toolbox of compounds to study this important serine protease and represents a step-change toward the design of covalent inhibitors targeting plasma kallikrein. We acknowledge their complex synthesis and purification. However, we feel that these preliminary studies add to the toolbox of available covalent warheads for future serine protease inhibitor design.

## Supporting Information Available

Synthetic methods for the preparation of compounds **17**–**22**, **24**–**27**, and **29**–**32**. ^1^H NMR and LCMS trace of **20**. Compound dissociation assay methodology. NMR stability studies on **17b**. Molecular docking studies on **32**. (PDF)

## ABBREVIATIONS

B2R: type 2 bradykinin receptor
CAS: contact activation system
C1-INH: C1 inhibitor protein
HAE: Hereditary angioedema
FDA: U.S. Food and Drug Administration
FXIa: Factor XIa
FXIIa: Factor XIIa
GPCR: G Protein-Coupled Receptor
HK: High-molecular-weight kininogen
KKS: kallikrein–kinin system
PKa: plasma kallikrein
PPACK: l-Prolyl-l-phenylalanyl-l-arginine chloromethyl ketone
PK: Plasma prekallikrein
SAR: Structure–activity relationship
