# Supplementary material for: Discovery of α-Amidobenzylboronates as Highly Potent Covalent Inhibitors of Plasma Kallikrein
Source: ACS Med Chem Lett. 2024 Mar 28;15(4):501–9. doi: 10.1021/acsmedchemlett.3c00572 (PMC11017388; doi:10.1021/acsmedchemlett.3c00572)
Supplement: Supplementary file 1 — ml3c00572_si_001.pdf [file ml3c00572_si_001.pdf]

Supporting Information

Discovery of α-Amidobenzylboronates as Highly Potent Covalent Inhibitors of Plasma Kallikrein

Matthew Allison,[1] Rebecca L. Davie,[2] Adrian J. Mogg,[2] Sally L. Hampton,[2] Jonas Emsley,[1] Michael J. Stocks.[1]*

[1] Biodiscovery Institute, School of Pharmacy, University of Nottingham, Nottingham, NG7 2RD, United Kingdom

[2] KalVista Pharmaceuticals Limited, Salisbury, SP4 0BF, United Kingdom

*Corresponding Author: michael.stocks@nottingham.ac.uk

Contents: **Page**

S1: General experimental procedures – Synthetic chemistry S2

S2: Abbreviations S2

S3: Synthetic chemistry methods S3

S4: References S17

S5: Pharmacology: Material and Methods S18

S6: NMR stability study on compound **17b**. S18

S7: Docking studies on **32.** S20

General Experimental procedures

S1: Synthetic chemistry - no unexpected or unusually high safety hazards were encountered.

Materials and Methods. HPLC grade and analytical grade chemicals and solvents were purchased from the standard suppliers and were used without further purification. Sigma Aldrich supplied high-grade silica, 60 Å, 230-400 mesh, for flash chromatography, and deuterated solvents (Chloroform-d, Methanol-d_4, DMSO-d_6) were purchased from Sigma Aldrich. Reactions were monitored by thin-layer chromatography (TLC) on commercially available silica pre-coated aluminum-backed plates (Merck Kieselgel 60 F254). Visualization was under UV light (254 nm and 366 nm), and where necessary staining with ninhydrin or potassium permanganate dips. NMR spectra were recorded with a Bruker AV(III) 400 NMR spectrometer. ^{1}H NMR was recorded at 400.13 MHz, and 13C NMR was recorded at 101.6 MHz. Deuterated solvents used for the preparation of NMR samples were $CDCl_3$, MeOD-d_4, or DMSO-d_6. Chemical shifts (δ) are reported in ppm with reference to the chemical shift of the deuterated solvent. Coupling constants (J) are recorded in hertz, and the signal multiplicities are described by the following: s, singlet; d, doublet; t, triplet; q, quartet; brs, broad singlet; m, multiplet; dd, doublet of doublets; ddd, double doublet of doublets; dt, doublet of triplets; p, pentet. NMR data was processed using MestReNova version 10.0.2. For the analysis of reaction mixtures and isolated compounds a Shimadzu UFLCXR HPLC was used, equipped with a Biosystems MDS SCIEX API2000 ESI+ MS. The column was a Gemini 3 µm C18 110 Å, LC column 50 x 2 nm. As eluent, a mixture was used of MeCN and H_2O, containing 0.1% formic acid. Samples were run using a gradient of 1:19 v/v to 19:1 v/v over either 5 or 15 minutes, with a flow rate of 0.5 mL/min. UV absorption was detected at 254 nm and 220 nm. Preparative RP-HPLC was performed on a Waters 2767 sample manager coupled to Waters 2525 binary-gradient module and a Waters 2457 dual-wavelength absorbance detector. The column used was a Phenomenex Gemini-NX (5 µm, 110 Å, C18, 150 × 21 mm) at ambient temperature. The flow rate was 25 mL/min, and UV detection was at 254 nm. Mobile phases were solvent A, 0.1% TFA in water, and solvent B, acetonitrile, degassed by helium bubble and sonication, respectively. HRMS was done on a Bruker microTOF II mass spectrometer using electrospray ionization (ESI-TOF) operating in the positive mode. Adducts within errors of ±10 ppm were reported.

S2: Abbreviations

^{t}Bu – tert-butyl; BuLi – butyllithium; calcd. – calculated; δ – chemical shift; DIPEA – diisopropylethylamine; DMF – *N,N*-dimethylformamide; DMSO – dimethylsulfoxide; ESI – electrospray ionisation; EtOAc – ethyl acetate; HATU - hexafluorophosphate azabenzotriazole tetramethyl uranium; HRMS – high resolution mass spectrometry; *J* – NMR coupling constant; LCMS – liquid chromatography mass spectrometry; LiHMDS - lithium hexamethyldisilazane; M – molecular ion; MeCN – acetonitrile; mmol – millimoles; m/z – mass-to-charge ratio; NMR – nuclear magnetic resonance; THF – tetrahydrofuran; TOF – time of flight; UV - ultraviolet

S3: Synthetic Chemistry Methods

Representative General Procedure for Esterification of Boronic Acids

To a solution of boronic acid (4 mmol) and pinacol (4 mmol) in dichloromethane (10 mL) was added anhydrous $MgSO_4$ (0.8 mmol). The resulting solution was stirred at room temperature for 16 hours, after which, it was filtered, and the filtrate concentrated *in vacuo* to afford the boronic ester which was used without further purification.

2-(3-chlorophenyl)-4,4,5,5-tetramethyl-1,3,2-dioxaborolane (13a).

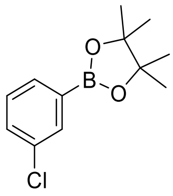

Colourless oil. (1430 mg, 99%) ^{1}H NMR (400 MHz, DMSO) δ 7.81 (d, *J* = 0.9 Hz, 1H), 7.75 (d, *J* = 6.8 Hz, 1H), 7.49 (dd, *J* = 6.8, 0.9 Hz, 1H), 7.34 (t, *J* = 6.8 Hz, 1H) 1.40 (s, 12H) ^{13}C NMR (101 MHz, DMSO) δ 137.1, 137.0, 134.3, 132.2, 131.9, 129.8, 85.0, 26.1 LCMS (ESI+) m/z calcd for $C_{12}H_{16}BClO_2$ = 238.1, found 239.1 [M+H]

2-(2-fluoro-4-methylphenyl)-4,4,5,5-tetramethyl-1,3,2-dioxaborolane (13b).

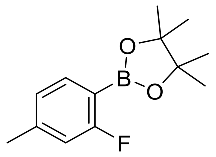

Colourless solid. (1.54 g, quant.) ^{1}H NMR (400 MHz, DMSO) δ 7.53 (s, 1H), 7.03 (d, *J* = 7.5 Hz, 1H), 6.97 (d, *J* = 7.5 Hz, 1H), 2.30 (s, 3H), 1.29 (s, 12H). ^{13}C NMR (101 MHz, DMSO) δ 168.2, 165.8, 145.1, 136.9, 125.3, 116.3, 84.0, 25.1, 21.4 LCMS (ESI+) m/z calcd. for $C_{13}H_{18}BFO_2$ = 236.1, found 237.1 [M+H].

Representative General Procedure for Preparation of Aminoboronate HCl salts:

To a -78ºC cooled solution of dichloromethane (33.04 mmol) and aryl pinacol boronate (6.61 mmol) in THF (20 mL) was added *n*-butyl lithium (6.61 mmol), the reaction mixture was allowed to stir for 16h, during which it warmed to ambient temperature. The reaction mixture was then re-cooled to -78°C and was allowed to stir for 24h, after which, the solvent was removed in vacuo, the residue was dissolved in hexane (50 mL) and passed through celite. The resulting filtrate was concentrated in vacuo and redissolved in diethyl ether (15 mL). This solution was then stirred at -78°C before careful addition of hydrochloric acid (10 mL, 19.82 mmol) (2M in diethyl ether). The solution was allowed to stir for 30 min and then stood at rt for 2h, leading to precipitation of the title compound which was washed by decantation with cold Et_2O and collected by filtration.

(3-Chlorophenyl)(4,4,5,5-tetramethyl-1,3,2-dioxaborolan-2-yl)methanamine hydrochloride (15a).

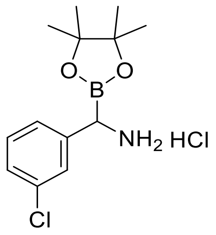

White solid. (509 mg, 40%, 3 steps) ^{1}H NMR (400 MHz, DMSO) δ 8.55 (s, 3H), 7.46 – 7.43 (m, 3H), 4.06 – 3.90 (m, 1H), 1.26 – 1.13 (m, 12H). ^{13}C NMR (101 MHz, DMSO); δ 135.2, 132.9, 130.5, 130.1, 129.1, 85.3, 66.8, 24.9, 24.8 LCMS was not obtained due to compound decomposition on LC column.

(2-Fluoro-4-methylphenyl)(4,4,5,5-tetramethyl-1,3,2-dioxaborolan-2-yl)methanamine hydrochloride (15b).

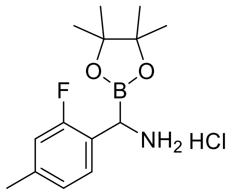

White solid. (250 mg, 20%, 3 steps) ^{1}H NMR (400 MHz, DMSO) δ 8.46 (s, 2H), 7.69 – 7.45 (m, 1H), 7.45 – 7.32 (m, 1H), 7.12 – 6.93 (m, 1H), 2.30 (s, 3H), 1.71 – 0.78 (m, 12H). ^{13}C NMR (101 MHz, DMSO) δ 161.46, 159.02, 141.11 (d), 131.17 (d), 125.77, 120.15, 120.00, 116.43, 116.22, 85.32, 24.84, 21.03. LCMS was not obtained due to compound decomposition on LC column.

<u>Representative General Procedure for Amide Coupling of Aminoboronate HCl salts:</u>

To a stirring, ice-cooled solution of aminoboronate salt (0.17 mmol), carboxylic acid (0.17 mmol) and HATU (0.19 mmol) in MeCN (2 mL) was added DIPEA (0.51 mmol). The ice-bath was removed and the reaction mixture was stirred at rt for a period of 3 hours. Upon completion, the solvent was removed *in vacuo* and the residue was purified by C18-prep-HPLC (H_2O/MeCN) and lyophilised to dryness to afford the title compounds.

1-Benzyl-N-((3-chlorophenyl)(4,4,5,5-tetramethyl-1,3,2-dioxaborolan-2-yl)methyl)-1H-pyrazole-4-carboxamide (17a).

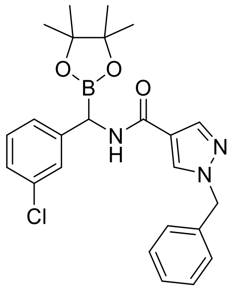

White solid (86 mg, 56%) ^{1}H NMR (400 MHz, MeOD); δ 8.44 (s, 1H), 8.13 (s, 1H), 7.43 – 7.31 (m, 6H), 7.19 – 7.12 (m, 2H), 7.09 – 7.03 (m, 1H), 5.44 (s, 2H), 5.38 (s, 1H), 3.3. (s, 1H), 1.29 – 0.96 (m, 12H) ^{13}C NMR (101 MHz, MeOD) δ 168.0, 143.9, 139.9, 138.7, 135.6, 133.6, 131.5,

129.1, 128.6, 128.1, 127.7, 127.5, 125.9, 125.2, 124.5, 109.9, 80.3, 55.9, 24.0, 23.5·LCMS (ESI+) m/z calcd. for $C_{24}H_{27}BClN_3O_3$ = 451.1, found 452.2 [M+H].

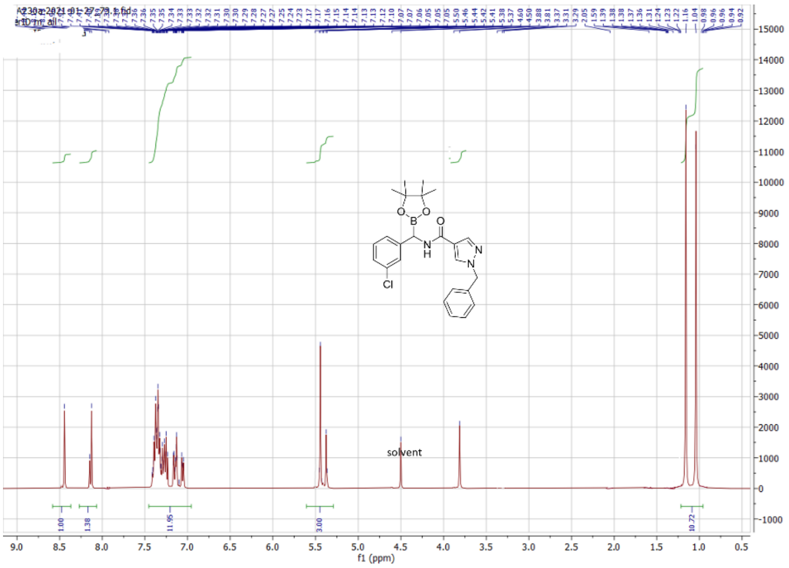

1-Benzyl-N-((2-fluoro-4-methylphenyl)(4,4,5,5-tetramethyl-1,3,2-dioxaborolan-2-yl)methyl)-1H-pyrazole-4-carboxamide (17b).

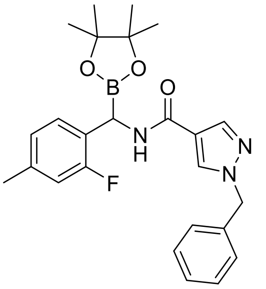

White solid (37 mg, 41%). 1H NMR (400 MHz, DMSO) δ 8.59 (s, 1H), 8.12 (s, 1H), 7.44 (m, 5H), 7.02 (m, 3H), 5.43, (s, 2H), 3.80 (s, 1H), 2.25 (s, 3H), 0.99 (s, 6H), 0.90 (s, 6H) 13C NMR (101 MHz, DMSO) δ 167.3, 158.8, 140.3, 136.8, 134.2, 129.2, 128.5, 128.4, 126.7 (d), 124.8 (d), 115.6, 115.4, 110.4, 79.6, 55.7, 25.5, 25.2, 20.8. LCMS (ESI+) m/z calcd. for C25H29BFN3O3 = 449.2, found 450.4 [M+H].

((1-Benzyl-1H-pyrazole-4-carboxamido)(3-chlorophenyl)methyl)boronic acid (18)·

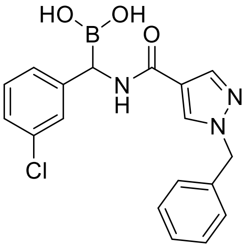

17 (50 mg, 0.11 mmol) and pentylboronic acid (63 mg, 0.55 mmol) were stirred in methanol/cyclohexane (1:1) (4 mL). 3N aqueous HCl (2 mL) was added, and the mixture was stirred at room temperature for 16 hours. Upon completion, the reaction mixture was washed

with cyclohexane (3 x 5 mL) and the water/methanol layer was collected and concentrated *in vacuo* at room temperature. The resulting residue was dissolved in 2N aqueous NaOH (2 mL) and re-concentrated *in vacuo* at room temperature. The remaining material was dissolved in DMSO (1 mL) and loaded onto a 12g Puriflash C18 cartridge and purified, eluting with 10 – 90% acetonitrile in water. The resulting aqueous fractions were lyophilised to dryness to afford the title compound as a white solid (9 mg, 23%). ^{1}H NMR (400 MHz, DMSO) δ 9.11 (s, 1H), 8.84 (dd, *J* = 4.5, 1.4 Hz, 1H), 8.74 (dd, *J* = 8.4, 1.4 Hz, 1H), 8.38 (s, 1H), 7.67 (dd, *J* = 8.4, 4.5 Hz, 1H), 7.42 – 7.32 (m, 6H), 5.51 (s, 2H), 5.41 – 5.33 (m, 1H) ^{13}C NMR (101 MHz, DMSO) δ 158.8, 153.2, 142.7, 136.9, 136.5, 134.9, 134.3, 130.4, 129.2, 129.1, 128.6, 128.5, 122.3, 107.1, 56.0, 55.5. LCMS was not obtained due to compound decomposition on HPLC column.

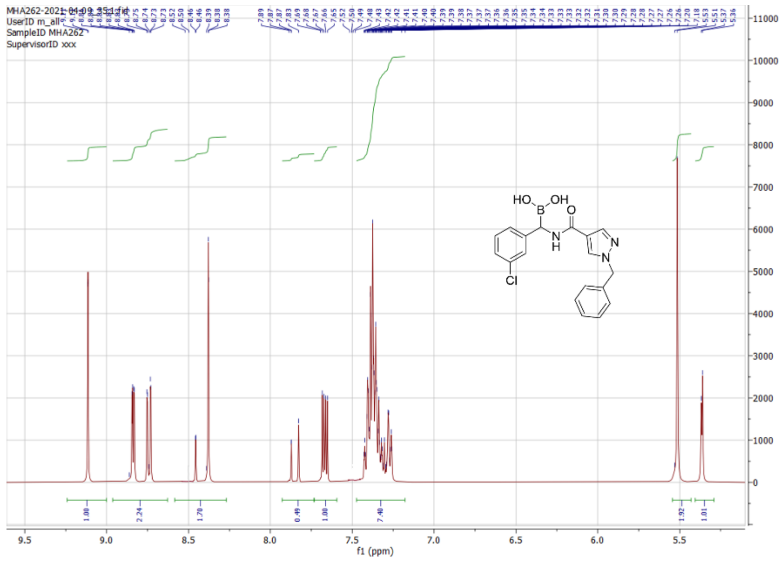

1-(4-((1H-pyrazol-1-yl)methyl)benzyl)-N-((3-chlorophenyl)(4,4,5,5-tetramethyl-1,3,2-dioxaborolan-2-yl)methyl)-1H-pyrazole-4-carboxamide (19).

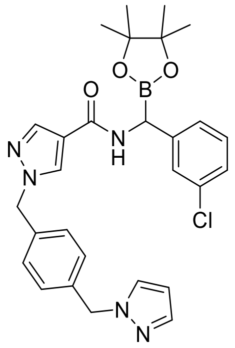

White solid (6 mg, 23%). 1H NMR (400 MHz, MeOD) δ 8.45 (s, 1H), 8.14 (s, 1H), 7.72 (s, 1H), 7.55 (s, 1H), 7.31 - 7.37 (m, 3H), 7.23 - 7.29 (m, 4H), 7.11 - 7.20 (m, 2H), 7.03 - 7.09 (m, 1H), 6.37 (d, *J* = 2.3 Hz, 1H), 5.45 (s, 2H), 5.39 (s, 2H), 3.83 (s, 1H), 1.17 (s, 6H), 1.05 (s, 6H). LCMS (ESI+) m/z calcd. for $C_{28}H_{31}BClN_5O_3$ = 531.2, found 532.2 [M+H].

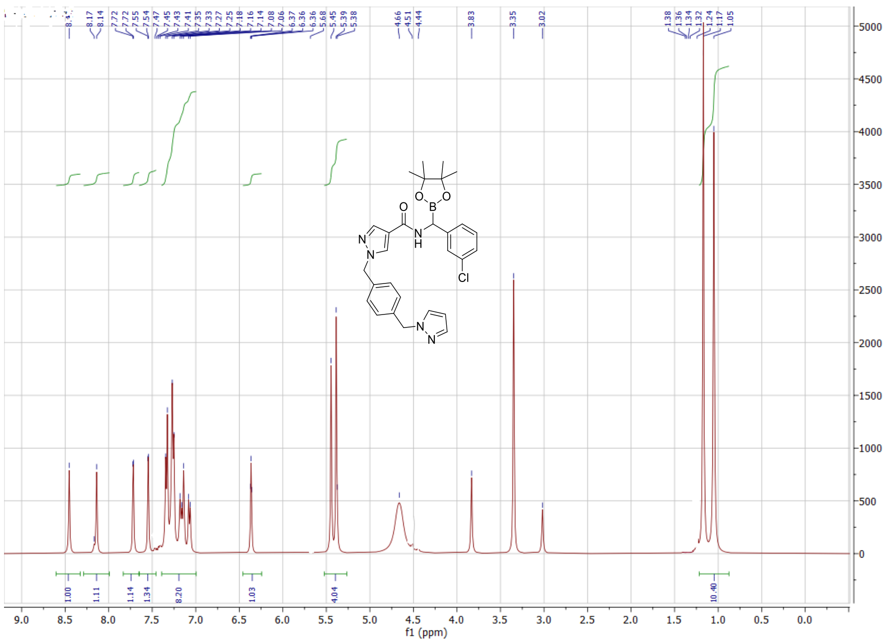

1-(4-((1H-Pyrazol-1-yl)methyl)benzyl)-N-((2-fluoro-4-methylphenyl)(4,4,5,5-tetramethyl-1,3,2-dioxaborolan-2-yl)methyl)-1H-pyrazole-4-carboxamide (20).

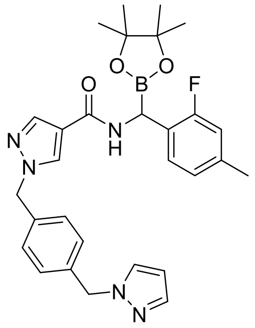

White solid (15 mg, 22%). ^{1}H NMR (400 MHz, DMSO, Amide N-H not observed.) δ 8.58 (s, 1H), 8.11 (s, 1H), 7.80 (d, J = 2.1 Hz, 1H), 7.45 (d, J = 2.1 Hz, 1H), 7.24 - 7.29 (m, 2H), 7.18 - 7.23 (m, 2H), 6.70 - 6.97 (m, 3H), 6.26 (t, J = 2.1 Hz, 1H), 5.41 (s, 2H), 5.31 (s, 2H), 3.79 (s, 1H), 2.26 (s, 3H), 0.99 (s, 6H), 0.90 (s, 6H). ^{13}C NMR (101 MHz, DMSO) δ 167.3, 140.3, 139.5, 138.0, 136.7, 136.6, 136.1, 134.3, 133.7, 130.6, 128.6, 128.3, (d) 126.8, 124.8, 124.7 (d), 110.5, 105.9, 103.9, 79.5, 76.0, 55.4, 54.8, 25.6, 25.3. ^{19}F NMR (377 MHz, DMSO) δ -119.7 (dd, *J* = 6.7, 12.0 Hz). ·LCMS (ESI+) m/z calcd for $C_{29}H_{33}BFN_5O_3$ = 529.3, found 530.3 [M+H]. HRMS m/z calcd. for [$C_{29}H_{34}BFN_5O_3$], 530.2739 [M+H], found 530.2744.

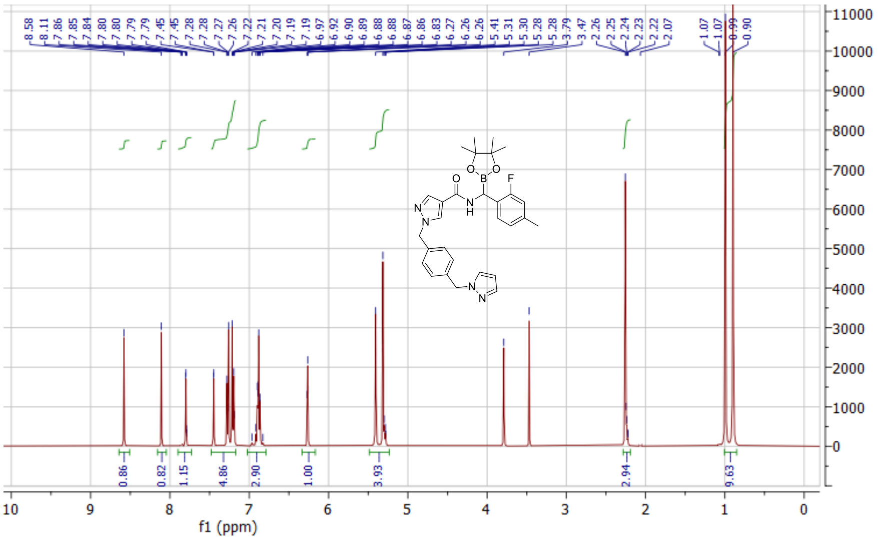

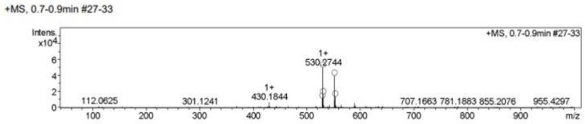

#	m/z	I %
1	112.0625	4.5
2	430.1844	13.8
3	443.2210	4.0
4	465.1986	4.3
5	517.2587	4.2
6	529.2749	23.3
7	530.2744	100.0
8	531.2763	32.9
9	532.2795	6.3
10	539.2365	4.2
11	542.2336	5.8
12	551.2590	18.7
13	552.2563	80.3
14	553.2589	26.8
15	554.2495	5.8
16	564.2173	8.5
17	589.3231	11.9
18	590.3246	4.9
19	610.1991	4.5
20	641.3305	3.9

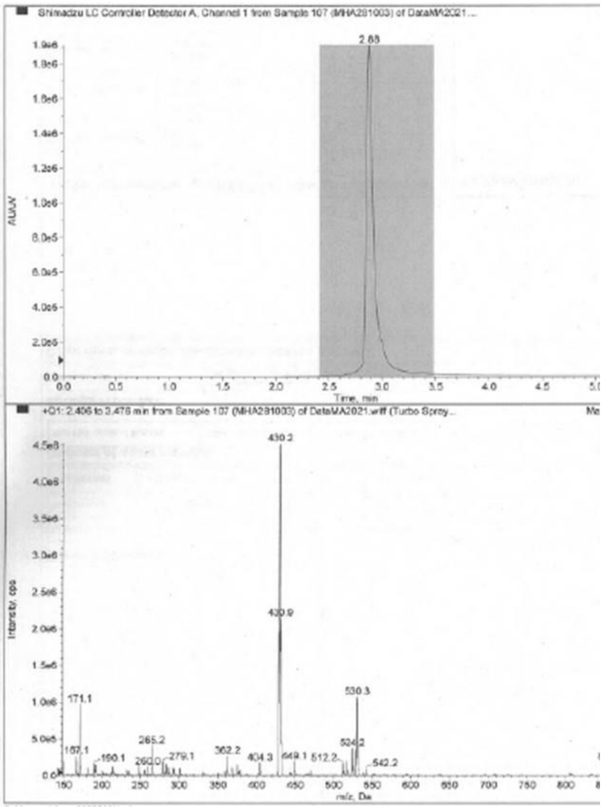

N-((2-Fluoro-4-methylphenyl)(4,4,5,5-tetramethyl-1,3,2-dioxaborolan-2-yl)methyl)-1-(4-((2-oxopyridin-1(2H)-yl)methyl)benzyl)-1H-pyrazole-4-carboxamide (21).

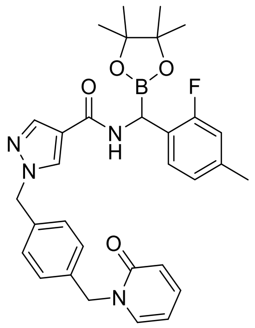

White solid, (2.5 mg, 3%). ^1H NMR (400 MHz, MeOD) δ 8.52 (dd, *J* = 1.4, 4.3 Hz, 2H), 8.18 (dd, *J* = 1.4, 8.4 Hz, 2H), 7.93 (s, 1H), 7.80 (s, 1H), 7.69 (dd, *J* = 2.1, 6.8 Hz, 1H), 7.53 (ddd, *J* = 2.1, 6.7, 8.9 Hz, 1H), 7.37 – 7.30 (m, 2H), 7.24 (d, *J* = 8.1 Hz, 2H), 6.58 (d, *J* = 9.2 Hz, 1H), 6.40 (td, *J* = 1.4, 6.7 Hz, 1H), 5.34 (s, 1H), 5.32 (s, 2H), 5.20 (s, 2H), 2.05 (s, 3H), 1.22 (s, 12H). ^{13}C NMR (101 MHz, MeOD) δ 168.9, 163.2, 147.2, 140.9, 140.7, 138.4, 136.5, 136.4, 135.0, 132.4, 128.0, 127.9, 127.8, 126.9, 122.1, 119.5 (d), 107.5, 74.4, 63.7, 54.8, 51.6, 23.6. LCMS (ESI+) m/z calcd. for $C_{31}H_{34}BFN_4O_4$ = 556.3, found 557.5 [M+H].

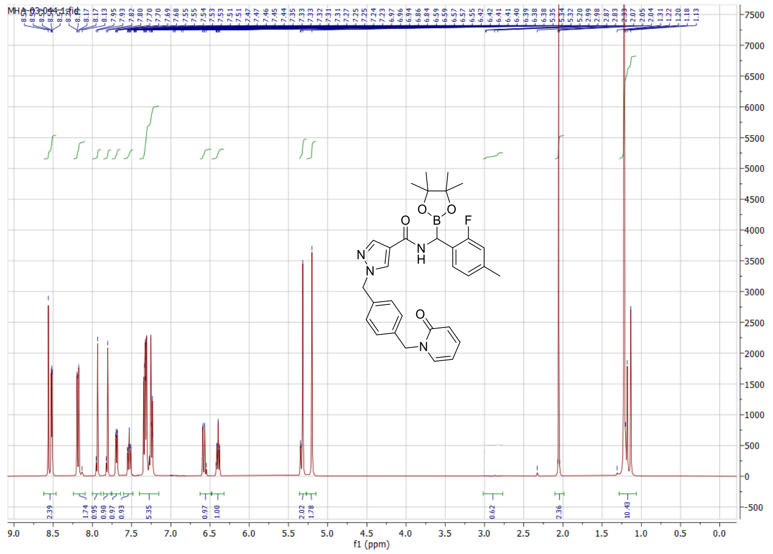

1-Benzyl-N-((2-fluoro-4-methylphenyl)(4,4,5,5-tetramethyl-1,3,2-dioxaborolan-2-yl)methyl)-1H-pyrazole-4-carboxamide (22).

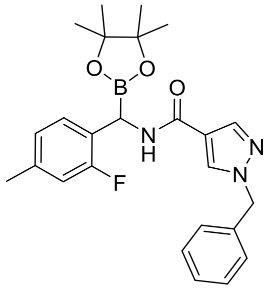

White solid (37 mg, 41%). ^1H NMR (400 MHz, DMSO, Amide N-H not observed.) δ 8.59 (s, 1H), 8.12 (s, 1H), 7.44 (m, 5H), 7.02 (m, 3H), 5.43, (s, 2H), 3.80 (s, 1H), 2.25 (s, 3H), 0.99 (s, 6H), 0.90 (s, 6H) ^{13}C NMR (101 MHz, DMSO) δ 167.3, 158.8, 140.3, 136.8, 134.2, 129.2, 128.5, 128.4, 126.7 (d), 124.8 (d), 115.6, 115.4, 110.4, 79.6, 55.7, 25.5, 25.2, 20.8. LCMS (ESI+) m/z calcd. for $C_{25}H_{29}BFN_3O_3$ = 449.33, found 450.4 [M+H].

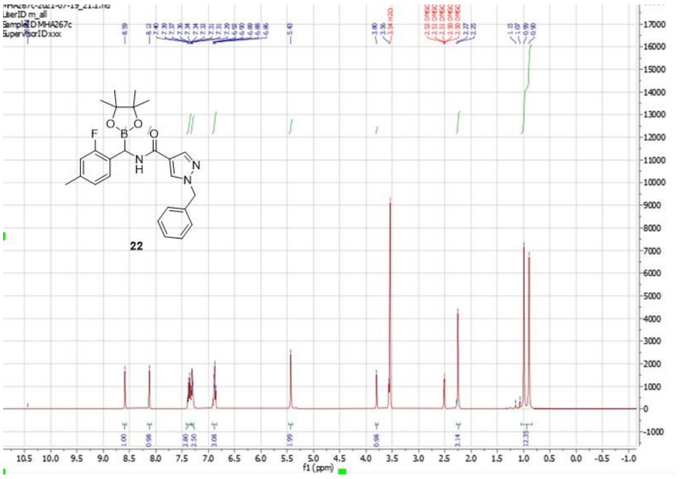

Representative General Procedure for Amide Coupling of benzylamines

To a stirring solution of carboxylic acid (0.16 mmol), HATU (0.16 mmol) and DIPEA (0.48 mmol) in MeCN (2 mL) was added substituted benzylamine (0.16 mmol). The reaction mixture was allowed to stir at room temperature for 1 hour, after which the solvent was removed *in vacuo* and the crude residue was purified by flash chromatography (0-10% MeOH in DCM) to afford the title compounds.

1-(4-((1H-pyrazol-1-yl)methyl)benzyl)-*N*-(3-chlorobenzyl)-1H-pyrazole-4-carboxamide (24).

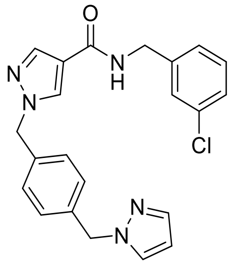

Off white solid (16 mg, 67%). ^{1}H NMR (400 MHz, DMSO) δ 8.65 (t, J = 6 Hz, 1H), 8.25 (d, *J* = 0.8 Hz, 1H), 7.89 (d, *J* = 0.8 Hz, 1H), 7.80 (dd, *J* = 0.7, 2.1 Hz, 1H), 7.44 (dd, *J* = 0.8, 1.9 Hz, 1H), 7.39 – 7.27 (m, 3H), 7.24 – 7.21 (m, 2H), 7.19 (d, *J* = 8.2 Hz, 2H), 6.25 (t, *J* = 2.1 Hz, 1H), 5.32 (s, 2H), 5.31 (s, 2H), 4.43 – 4.36 (m, 2H). ^{13}C NMR (101 MHz, DMSO) δ 161.14, 141.86, 138.38, 138.20, 136.81, 135.53, 132.31, 130.97, 129.58, 129.51, 127.43, 127.16, 126.42, 126.06, 125.32, 117.76, 104.85, 59.15, 54.13, 53.68. LCMS (ESI+) m/z calcd. for $C_{22}H_{20}ClN_5O$ = 406.1, found 406.1 [M+H].

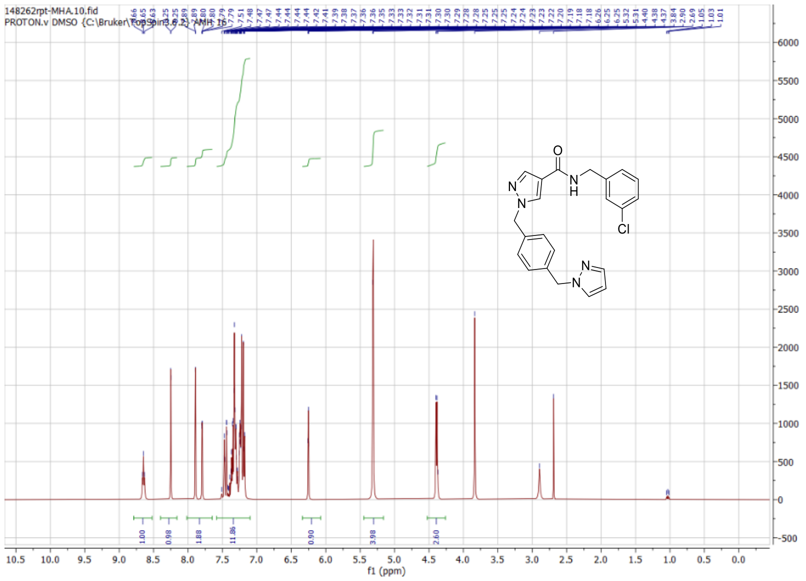

N-(3-Chlorobenzyl)-1-(4-((2-oxopyridin-1(2H)-yl)methyl)benzyl)-1H-pyrazole-4-carboxamide (25).

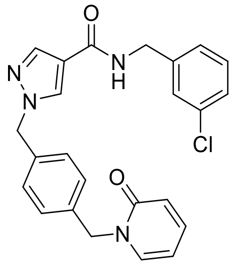

White solid (100 mg, 89%) ^{1}H NMR (400 MHz, DMSO) δ 8.66 (t, *J* = 6.0 Hz, 1H), 8.26 (d, *J* = 0.8 Hz, 1H), 7.90 (d, *J* = 0.7 Hz, 1H), 7.76 (ddd, *J* = 6.8, 2.1, 0.7 Hz, 1H), 7.41 (ddd, *J* = 8.9, 6.6, 2.1 Hz, 1H), 7.37 – 7.30 (m, 2H), 7.29 – 7.27 (m, 2H), 7.27 – 7.21 (m, 4H), 6.40 (ddd, *J* = 9.1, 1.4, 0.7 Hz, 1H), 6.22 (td, *J* = 6.7, 1.4 Hz, 1H), 5.32 (s, 2H), 5.07 (s, 2H), 4.39 (d, *J* = 6.0 Hz, 2H). ^{13}C NMR (101 MHz, DMSO) δ 161.2, 160.8, 141.9, 139.5, 138.5, 138.2, 136.5, 135.5, 132.3, 131.0, 129.6, 127.5, 127.3, 126.4, 126.1, 125.3, 119.3, 117.8, 104.9, 54.1, 50.2, 40.8. LCMS (ESI+) m/z calcd. for $C_{24}H_{21}ClN_4O_2$ = 432.1, found 433.2 [M+H].

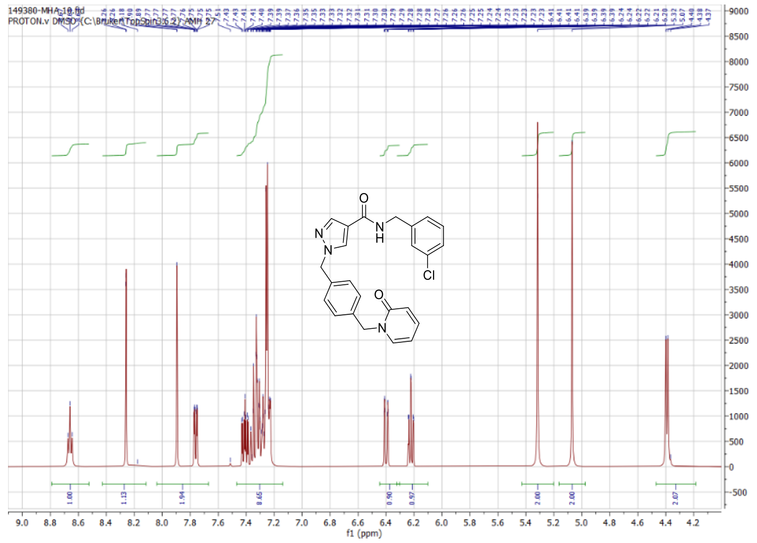

1-(4-((1H-Pyrazol-1-yl)methyl)benzyl)-*N*-(2-fluoro-4-methylbenzyl)-1H-pyrazole-4-carboxamide (26).

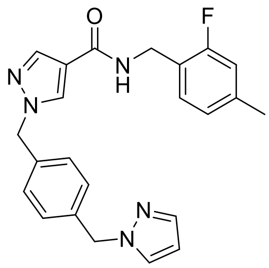

White solid (8 mg, 34%) ^{1}H NMR (400 MHz, DMSO) ^{1}H NMR (400 MHz, DMSO) δ 8.54 (s, 1H), 8.25 (s, 1H), 7.89 (s, 1H), 7.80 (d, *J* = 2.3 Hz, 1H), 7.44 (d, *J* = 1.8 Hz, 2H), 7.29 – 7.15 (m, 4H), 7.05 – 6.91 (m, 2H), 6.25 (t, *J* = 2.1 Hz, 1H), 5.45 – 5.11 (m, 4H), 4.38 (d, *J* = 5.9 Hz, 2H), 2.28 (s, 3H). ^{13}C NMR (101 MHz, DMSO) δ 162.1, 139.5, 139.3, 137.9, 136.6, 132.0, 130.6 (d), 129.9, 129.9, 128.5, 128.2, 125.3, 125.3, 123.6, 123.4, 118.9, 116.0, 115.8, 105.9, 56.5, 55.2, 54.8. LCMS (ESI+) m/z calcd. for $C_{23}H_{22}FN_5O$ = 403.2, found 404.1 [M+H].

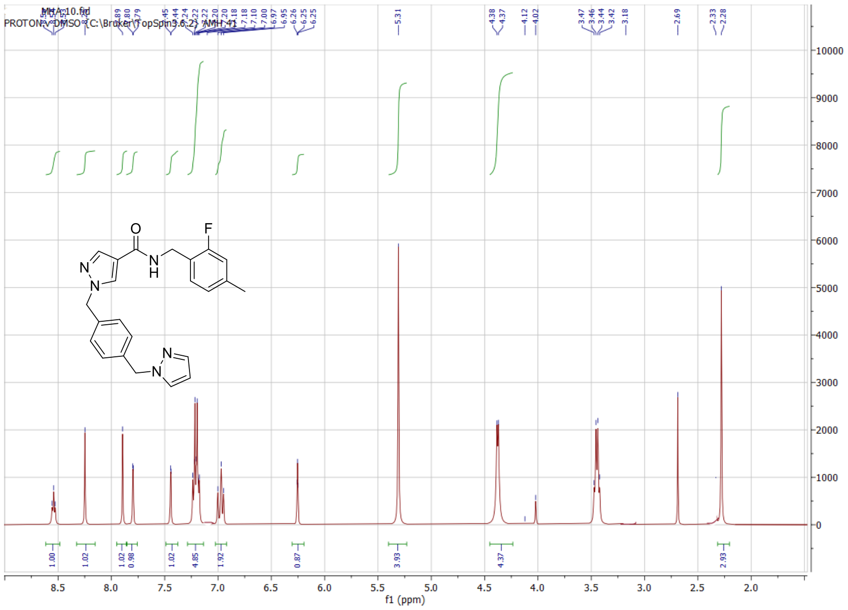

N-(2-fluoro-4-methylbenzyl)-1-(4-((2-oxopyridin-1(2H)-yl)methyl)benzyl)-1H-pyrazole-4-carboxamide (27).

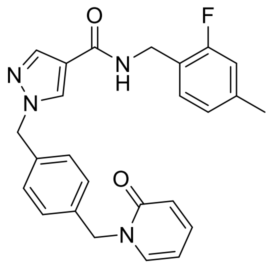

Off white solid (88 mg, 83%) ^{1}H NMR (400 MHz, DMSO) δ 8.53 (t, *J* = 5.8 Hz, 1H), 8.25 (s, 1H), 7.89 (s, 1H), 7.76 (dd, *J* = 6.8, 2.1 Hz, 1H), 7.41 (ddd, *J* = 8.9, 6.6, 2.1 Hz, 1H), 7.33 – 7.18 (m, 4H), 7.01 – 6.87 (m, 2H), 6.40 (d, *J* = 9.2 Hz, 1H), 6.22 (td, *J* = 6.7, 1.4 Hz, 1H), 5.31 (s, 2H), 5.07 (s, 2H), 4.37 (d, *J* = 5.8 Hz, 2H), 2.28 (s, 3H). ^{13}C NMR (101 MHz, DMSO) δ 161.1, 160.8, 139.5,

138.5, 138.2, 138.1, 136.5, 135.5, 130.9 (d), 128.8, 128.8, 127.5, 127.3, 124.2, 122.5, 122.4, 119.3, 117.8, 114.9, 114.7, 104.9, 54.1, 50.2, 34.9, 19.9. LCMS (ESI+) m/z calcd. for $C_{25}H_{23}FN_4O_2$ = 430.2, found 431.1 [M+H].

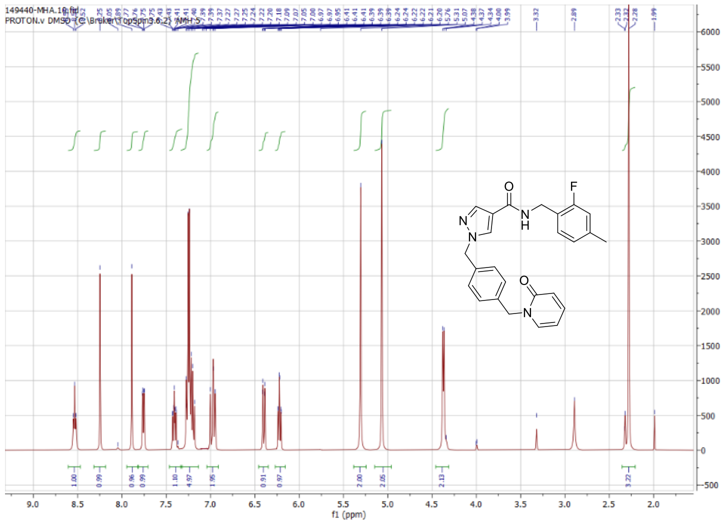

1-(1-(4-((1H-pyrazol-1-yl)methyl)benzyl)-1H-pyrazol-4-yl)ethan-1-one (28).

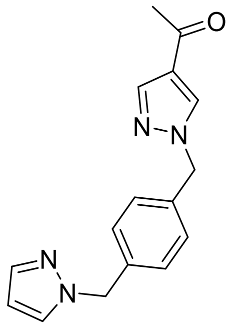

To an ice-cooled, stirring solution of 1-(4-(bromomethyl)benzyl)-1H-pyrazole (500 mg, 2.0 mmol) and 1-(1H-pyrazol-4-yl)ethan-1-one (264 mg, 2.4 mmol) in THF (15 mL) was added NaH (60% dispersion in mineral oil) (120 mg, 3.0 mmol). The solution was heated to 50 ^{o}C for a period of 3 hours, after which it was concentrated *in vacuo* and partitioned between EtOAc and water. The aqueous layer was extracted with EtOAc (3 x 20 mL), the organics were combined, washed with brine, dried over $MgSO_4$, filtered, and concentrated *in vacuo*. The resulting residue was purified by flash column chromatography (30 – 50 % EtOAc in cyclohexane) to afford the title compound. White solid (353 mg, 63%). ^{1}H NMR (400 MHz, DMSO) δ 8.51 (s, 1H), 7.92 (s, 1H), 7.80 (d, *J* = 2.2 Hz, 1H), 7.45 (d, *J* = 1.8 Hz, 1H), 7.25 (d, *J* = 8.0 Hz, 2H), 7.19 (d, *J* = 8.0 Hz, 2H), 6.26 (t, *J* = 2.1 Hz, 1H), 5.34 (s, 2H), 5.31 (s, 2H), 2.35 (s, 3H). ^{13}C NMR (101 MHz, DMSO) δ 192.0, 140.6, 139.5, 138.0, 136.4, 134.0, 132.0, 131.9, 130.6, 124.3, 105.9, 55.2, 54.8, 28.3. LCMS (ESI+) m/z calcd. for $C_{16}H_{16}N_4O$ = 280.1, found 281.1 [M+H].

(E)-1-(1-(4-((1H-pyrazol-1-yl)methyl)benzyl)-1H-pyrazol-4-yl)-3-(3-chlorophenyl)prop-2-en-1-one (29).

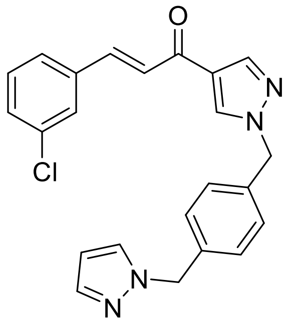

1-(1-(4-((1H-pyrazol-1-yl)methyl)benzyl)-1H-pyrazol-4-yl)ethan-1-one (100 mg, 0.36 mmol), 3-chlorobenzaldehyde (51 mg, 0.04 mLm 0.36 mmol) and NaOH (29 mg, 0.72 mmol) were dissolved in MeOH. The mixture was allowed to stir at room temperature for 16 hours, after which the reaction mixture was concentrated *in vacuo* and the residue partitioned between EtOAc and water. The aqueous layer was extracted with EtOAc (3 x 20 mL), the organics were combined, washed with brine, dried over $MgSO_4$, filtered, and concentrated *in vacuo*. The resulting residue was purified by flash column chromatography (30 – 50 % EtOAc in cyclohexane) to afford the title compound. White solid (117 mg, 81%). ^{1}H NMR (400 MHz, DMSO) δ 8.77 (s, 1H), 8.20 (s, 1H), 8.04 – 7.96 (m, 1H), 7.80 (d, *J* = 2.0 Hz, 1H), 7.79 – 7.74 (m, 1H), 7.65 (d, *J* = 8.7 Hz, 2H), 7.52 – 7.47 (m, 2H), 7.44 (d, *J* = 2.0 Hz, 1H), 7.27 (d, *J* = 8.0 Hz, 2H), 7.21 (d, *J* = 8.0 Hz, 2H), 6.26 (t, *J* = 2.0 Hz, 1H), 5.38 (s, 2H), 5.32 (s, 2H). ^{13}C NMR (101 MHz, $CDCl_3$) δ 188.5, 145.3, 139.7, 139.5, 137.1, 135.5, 130.9, 129.5, 129.3, 128.3, 128.2, 125.3, 124.0, 124.0, 119.4, 117.0, 116.8, 106.1, 67.8, 56.0, 55.5. LCMS (ESI+) m/z calcd for $C_{23}H_{19}ClN_4O$ = 402.1, found 403.0 [M+H].

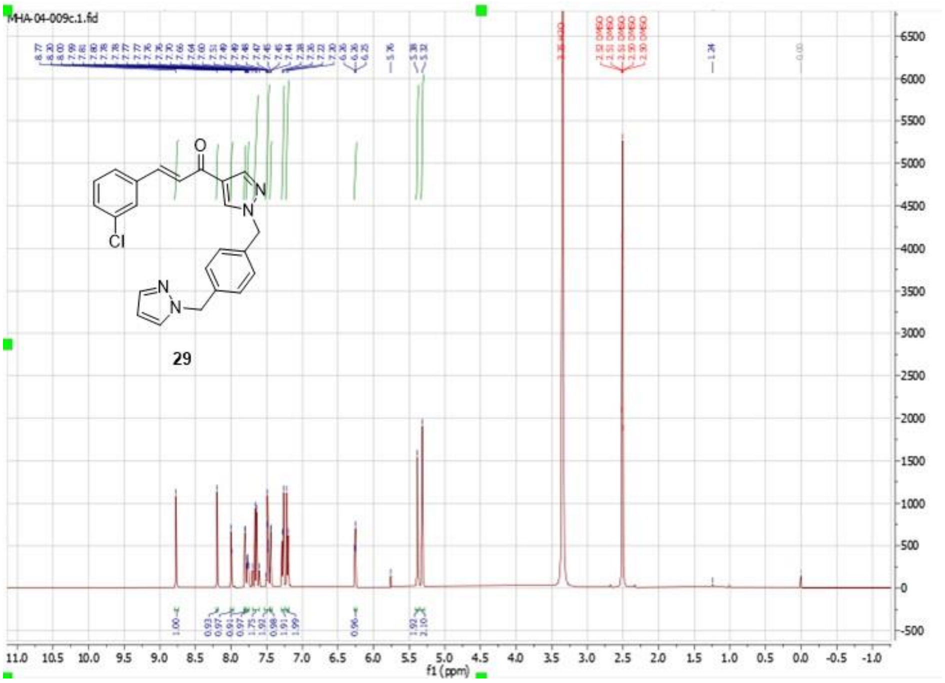

(E)-1-(1-(4-((1H-pyrazol-1-yl)methyl)benzyl)-1H-pyrazol-4-yl)-3-(2-fluoro-4-methylphenyl)prop-2-en-1-one (30).

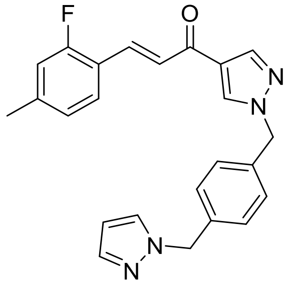

1-(1-(4-((1H-pyrazol-1-yl)methyl)benzyl)-1H-pyrazol-4-yl)ethan-1-one (100 mg, 0.36 mmol),2-fluoro-4-methylbenzaldehyde (50 mg, 0.04 mL, 0.36 mmol) and NaOH (29 mg, 0.72 mmol) were dissolved in MeOH. The mixture was allowed to stir at room temperature for 16 hours, after which the reaction mixture was concentrated *in vacuo* and the residue partitioned between EtOAc and water. The aqueous layer was extracted with EtOAc (3 x 20 mL), the organics were combined, washed with brine, dried over $MgSO_4$, filtered, and concentrated *in vacuo*. The resulting residue was purified by flash column chromatography (30 – 50 % EtOAc in cyclohexane) to afford the title compound. White solid (101 mg, 70%). ^{1}H NMR (400 MHz, $CDCl_3$) δ 8.08 (s, 1H), 8.00 (s, 1H), 7.57 (d, J = 2.1 Hz, 1H), 7.47 - 7.54 (m, 1H), 7.42 (d, J = 2.1 Hz, 1H), 7.28 (s, 2H), 7.26 (s, 1H), 7.25 – 7.23 (m, 2H), 7.21 (s, 1H), 7.01 (d, J = 7.7 Hz, 1H), 6.96 (d, J = 11.8 Hz, 1H), 6.31 (d, J = 2.1 Hz, 1H), 5.34 (s, 4H), 2.40 (s, 3H). ^{13}C NMR (101 MHz, $CDCl_3$) δ 183.5, 143.1, 143.0, 140.8, 139.8, 137.3, 136.3, 135.9, 134.9, 132.2, 129.7 (d), 129.4, 128.5, 128.2, 125.4 (d), 125.1, 124.84, 124.77, 116.9, 116.7, 106.1, 56.2, 55.4, 21.4. LCMS (ESI+) m/z calcd. for $C_{24}H_{21}FN_4O$ = 400.2, found 401.2 [M+H].

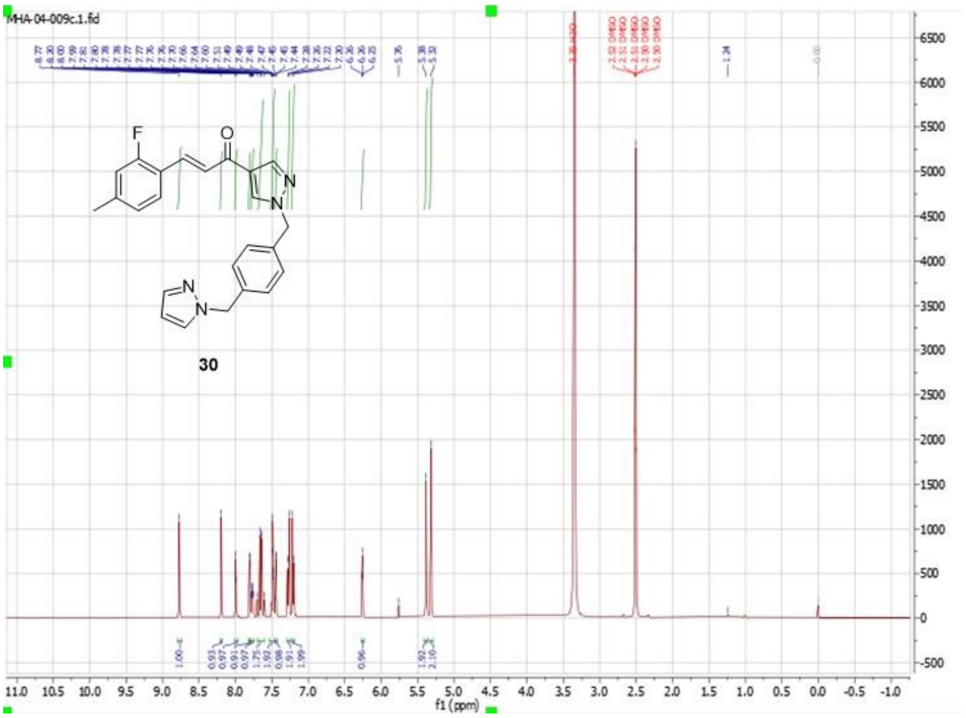

Representative general procedure for CuCl catalytic borylation of enones 29-30.

CuCl (0.5 mg, 0.004 mmol), DPEPhos (6 mg, 0.011 mmol) and NaO^{t}Bu (1.1 mg, 0.011 mmol) were added to a microwave vial which was sealed and purged with nitrogen. Anhydrous THF (0.5 mL) was added, and the suspension was stirred for 30 minutes. B_2Pin_2 (36 mg, 0.14 mmol)

in 0.5 mL anhydrous THF was then added, and the mixture was stirred for a further 10 minutes before addition of enone (50 mg, 0.12 mmol) and methanol (8 mg, 80 µL, 0.24 mmol) in 1 mL anhydrous THF. The reaction mixture was allowed to stir at room temperature for 16 hours under nitrogen, after which, it was filtered through celite, concentrated *in vacuo* and purified by silica gel flash chromatography (20 – 50 % EtOAc in cyclohexane) to afford the product as a colourless oil.

1-(1-(4-((1H-pyrazol-1-yl)methyl)benzyl)-1H-pyrazol-4-yl)-3-(3-chlorophenyl)-3-(4,4,5,5-tetramethyl-1,3,2-dioxaborolan-2-yl)propan-1-one (31).

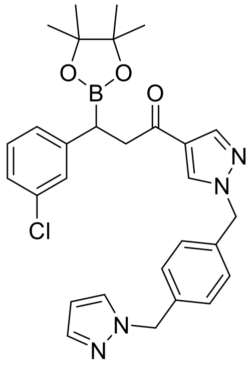

Colourless oil (35 mg, 56 %) ^{1}H NMR (400 MHz, MeOD) δ 8.31 (s, 1H), 7.97 (s, 1H), 7.68 (d, *J* = 2.3 Hz, 1H), 7.52 (d, *J* = 2.0 Hz, 1H), 7.29 – 7.26 (m, 2H), 7.26 – 7.22 (m, 2H), 7.22 – 7.17 (m, 4H), 7.17 – 7.12 (m, 2H), 6.33 (t, *J* = 2.1 Hz, 1H), 5.34 (s, 4H), 3.40 – 3.27 (m, 2H), 3.23 (dd, *J* = 5.6, 17.9 Hz, 1H), 2.67 (dd, *J* = 5.5, 10.3 Hz, 1H), 1.22 (s, 12H). ^{13}C NMR (101 MHz, MeOD) δ 194.5, 144.2, 140.0, 139.2, 137.2, 135.7, 133.8, 133.1, 130.3, 129.5, 128.0, 127.9, 127.6, 126.4, 125.3, 123.2, 105.6, 83.5, 82.7, 74.4, 55.2, 54.5, 43.2, 23.7, 23.5. LCMS (ESI+) m/z calcd. for $C_{29}H_{32}BClN_4O_3$ = 530.2, found 531.1 [M+H].

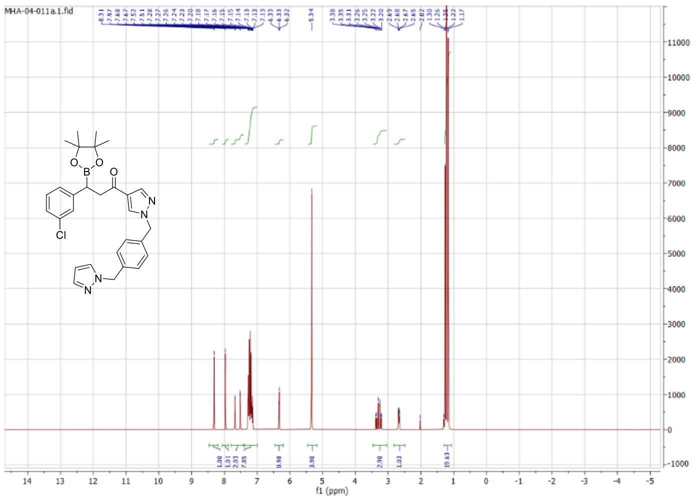

1-(1-(4-((1H-pyrazol-1-yl)methyl)benzyl)-1H-pyrazol-4-yl)-3-(2-fluoro-4-methylphenyl)-3-(4,4,5,5-tetramethyl-1,3,2-dioxaborolan-2-yl)propan-1-one (32).

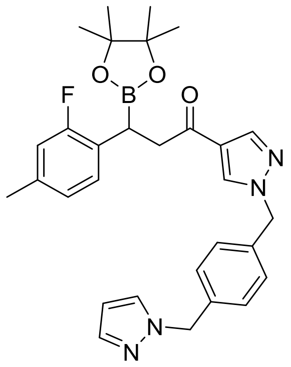

Colourless oil (22 mg, 34 %). ^{1}H NMR (400 MHz, MeOD) δ 8.28 (s, 1H), 7.93 (s, 1H), 7.69 (d, *J* = 2.3 Hz, 1H), 7.51 (d, *J* = 1.9 Hz, 1H), 7.29 (d, *J* = 8.1 Hz, 2H), 7.24 (d, *J* = 8.0 Hz, 3H), 7.21 – 7.19 (m, 1H), 6.88 – 6.82 (m, 2H), 6.33 (t, *J* = 2.2 Hz, 1H), 5.40 (s, 3H), 5.35 (s, 2H), 3.30 (d, *J* = 9.3 Hz, 1H), 3.15 (dd, *J* = 6.1, 17.6 Hz, 1H), 2.91 (dd, *J* = 6.1, 9.2 Hz, 1H), 2.28 (s, 3H), 1.31 – 1.18 (m, 12H). ^{13}C NMR (101 MHz, MeOD) δ 194.8, 139.9, 139.1, 137.6, 137.2, 135.7, 133.0, 130.3, 129.8 (d, *J* = 5.1 Hz), 127.9, 127.6, 125.3, 124.4 (d), 123.3, 115.3, 115.1, 105.6, 83.5, 74.4, 55.1, 54.5, 42.2, 23.6, 23.5 (d), 19.5. LCMS (ESI+) m/z calcd. for $C_{30}H_{34}BFN_4O_3$ = 528.3, found 529.3 [M+H].

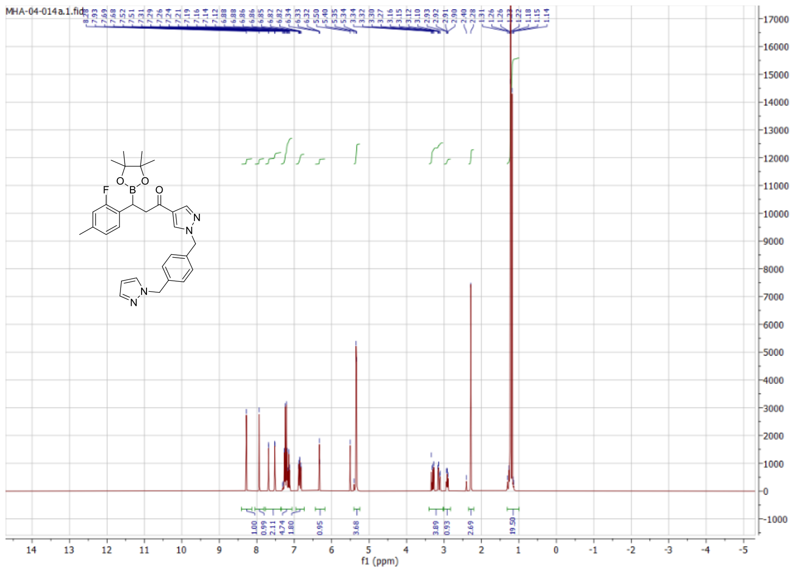

S4: References

(1) Liu, B.; Trout, R. E. L.; Chu, G. H.; Mcgarry, D.; Jackson, R. W.; Hamrick, J. C.; Daigle, D. M.; Cusick, S. M.; Pozzi, C.; De Luca, F.; Benvenuti, M.; Mangani, S.; Docquier, J. D.; Weiss, W. J.; Pevear, D. C.; Xerri, L.; Burns, C. J. Discovery of Taniborbactam (VNRX-5133): A Broad-Spectrum Serine- And Metallo-β-Lactamase Inhibitor for Carbapenem-Resistant Bacterial Infections. *J. Med. Chem.* **2020**.

(2) Hinkes, S. P. A.; Klein, C. D. P. Virtues of Volatility: A Facile Transesterification Approach to Boronic Acids. *Org. Lett.* **2019**.

(3) Mun, S.; Lee, J. E.; Yun, J. Copper-Catalyzed β-Boration of α,β-Unsaturated Carbonyl Compounds: Rate Acceleration by Alcohol Additives. *Org. Lett.* **2006**, *8* (21), 4887–4889.

S5: Pharmacology: Material and Methods:

Compound assays for activity against plasma kallikrein, FXIIa and FXIa. The assay conditions were previously described in Davie, R. L.; Edwards, H. J.; Evans, D. M.; Hodgson, S. T.; Stocks, M. J.; Smith, A. J.; Rushbrooke, L. J.; Pethen, S. J.; Roe, M. B.; Clark, D. E.; McEwan, P. A.; Hampton, S. L. Sebetralstat (KVD900): A Potent and Selective Small Molecule Plasma Kallikrein Inhibitor Featuring a Novel P1 Group as a Potential Oral On-Demand Treatment for Hereditary Angioedema. J. Med. Chem. 2022, 65(20):13629-13644

Compound Dissociation Experiments: All dissociation experiments were performed in buffer containing 100 mM Tris-HCl, 0.5 mM EDTA, 0.1% BSA pH 8. PKa enzyme (6.25 nM) and inhibitors were pre-incubated together using a concentration of inhibitor that generated a resulting EI complex ≥ 96% of the total enzyme concentration. After 10 min, the solution was rapidly diluted 62.5-fold. This resulted in an enzyme concentration of 0.1 nM and an inhibitor concentration well below the IC_{50} with the concentration of the EI complex approximately ≤ 10%-15%. This solution was immediately transferred to an assay plate containing the fluorescent substrate (Pro-Phe-Arg-AFC) at a concentration ~10x Km in order to limit re-entrance of the inhibitor after dissociation from the enzyme. Change in fluorescence signal was then monitored over 10 min.

S6: NMR stability study on compound 17b.

Compound **17b** was dissolved in pH7.4 phosphate buffered D_2O

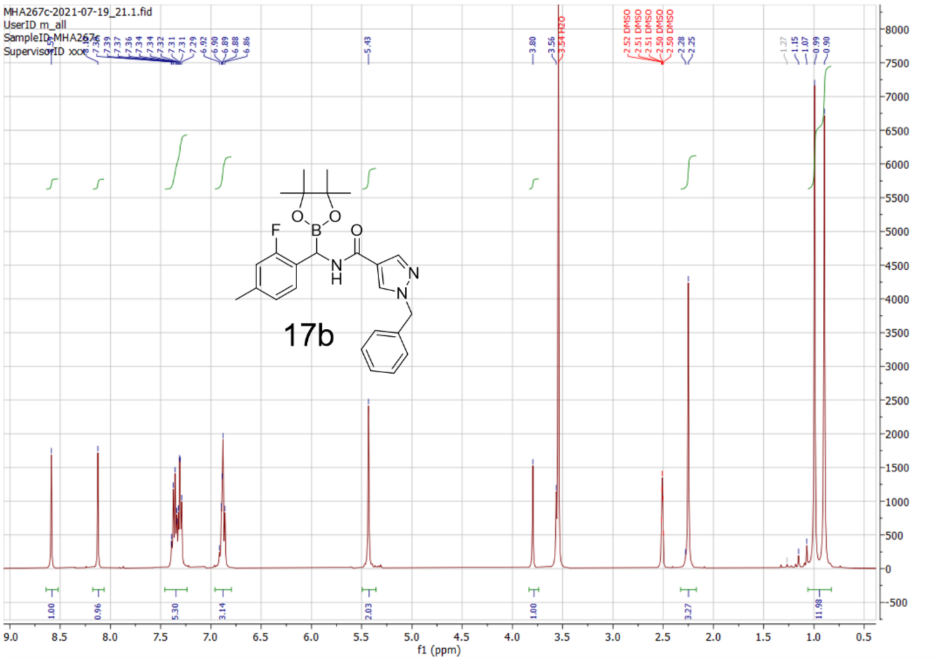

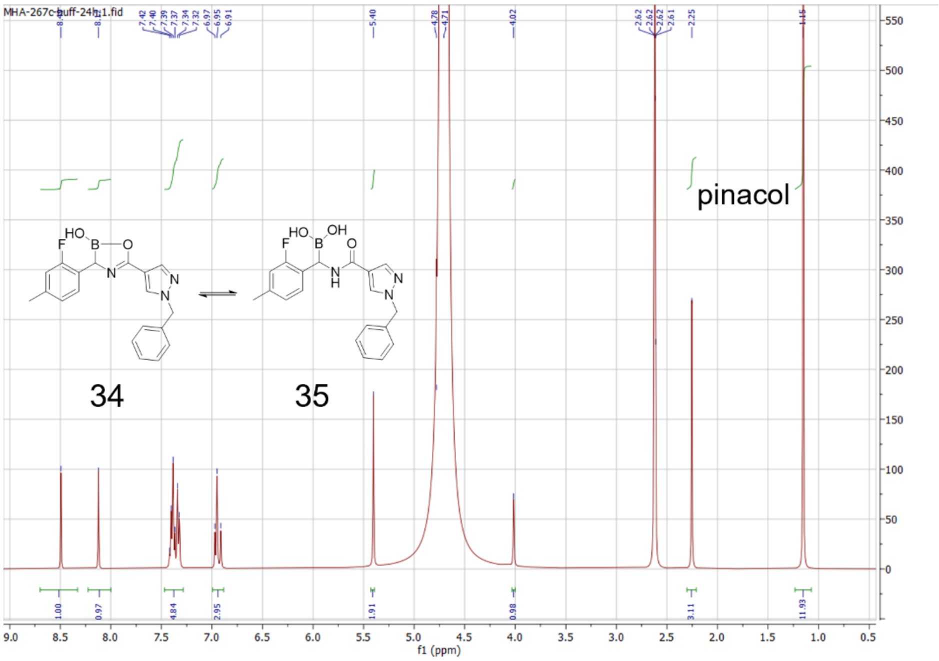

Figure S1: [A] ^{1}HNMR of **17b** in D6-DMSO; [B] ^{1}HNMR of **17b** in pH7.4 phosphate buffered D_2O + DCl. ^{1}HNMR analysis shows primarily compound **34** is observed.

LCMS (ESI+) m/z calcd. for $C_{19}H_{17}BFN_3O_2$ = 349.14, found 350.5 [M+H].

S7: Docking studies on compound 32.

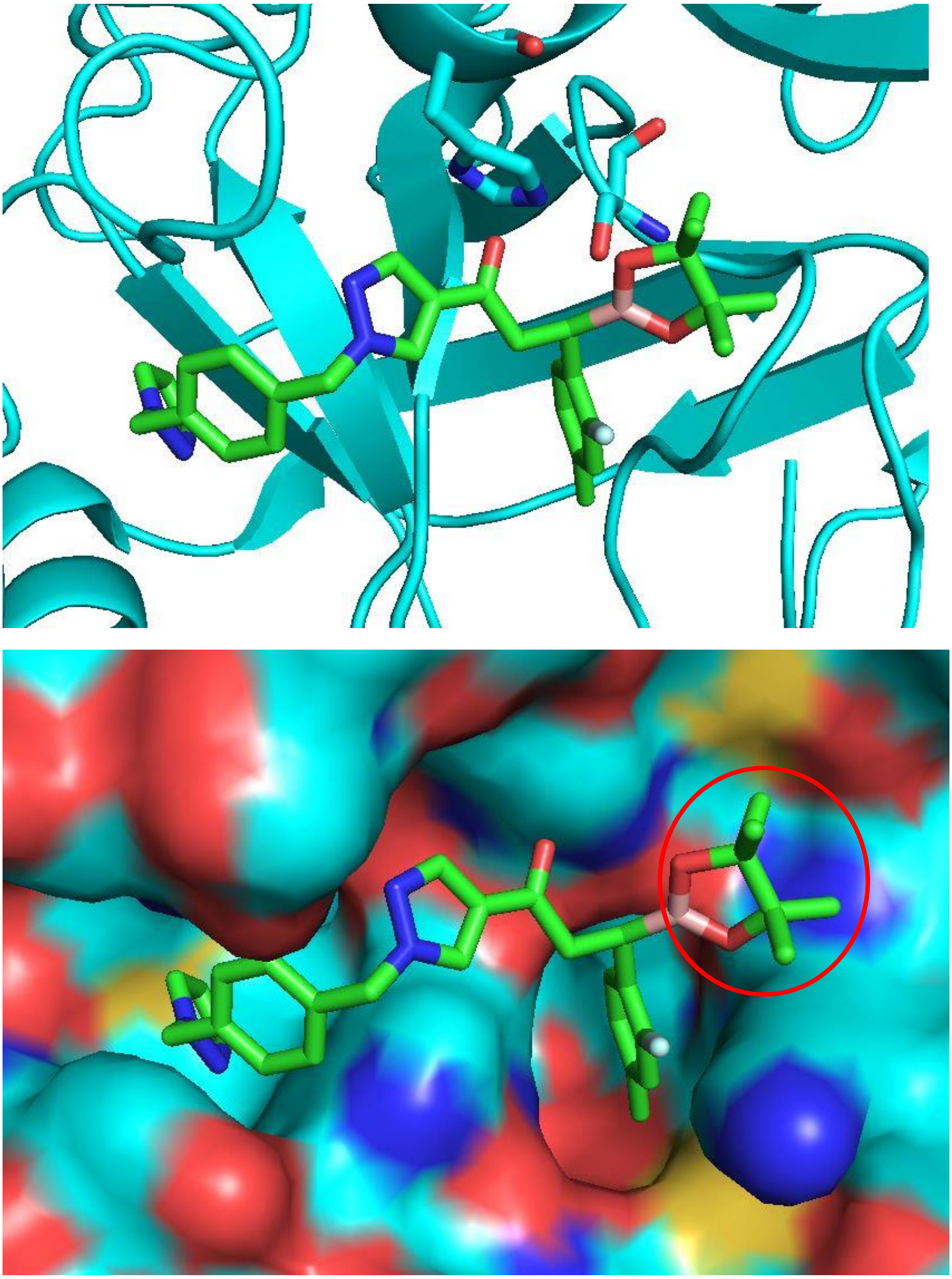

Figure S2 Docking of **32** into PKa active site (PDB6O1S). Docking experiments were performed using OEDOCKING Hybrid docking using the Ala-195 mutant. Docking poses were then visualized in PKa active site (PDB6O1S). Pinacol boronate highlighted showing exposure to solvent with no interaction with protein.
